# Cytotoxic T lymphocytes and their dual role in modulating blood-brain barrier integrity in immune-mediated neurological pathologies

**DOI:** 10.1186/s12967-025-07288-3

**Published:** 2025-12-23

**Authors:** Bin Li, Wen Xi, Ping Li

**Affiliations:** 1 Institute of Comparative Medicine, Jiangsu Co-innovation Center for Prevention and Control of Important Animal Infectious Diseases and Zoonoses, Yangzhou University, Yangzhou, China; 2 Department of Human Anatomy and Histoembryology, Nanjing University of Chinese Medicine, Nanjing, China; 3 Department of Spleen and Gastroenterology, Qinhuangdao Chinese Medicine Hospital, Beijing University of Chinese Medicine Dongfang Hospital, Qinhuangdao, China

**Keywords:** CD8^+^ CTLs, CD4^+^ CTLs, BBB, Neurodegenerative disease, Glioma, Infectious neurological disorder

## Abstract

The blood-brain barrier (BBB) is a dynamic, multicellular interface that preserves central nervous system (CNS) homeostasis by restricting entry of pathogens and circulating cells. Cytotoxic T lymphocytes (CTLs), comprising both CD8⁺ and CD4⁺ subsets, are central to adaptive immunity through targeted elimination of infected or transformed cells. However, in immune-mediated neurological disorders, including viral encephalitis, multiple sclerosis, Parkinson’s disease, and glioma, CTLs effector functions can inadvertently compromise BBB integrity. Here, we integrate findings from primary research to delineate three principal mechanisms by which CTLs modulate the BBB: (1) direct cytotoxicity, in which perforin/granzyme release and FasL-Fas interactions induce endothelial cell apoptosis; (2) proinflammatory cytokine signaling, notably IFN‑γ and TNF‑α activation of JAK/STAT and NF‑κB pathways in brain microvascular endothelial cells; and (3) chemokine‑driven leukocyte trafficking, wherein CXCL10 and CCL5 gradients promote CTLs and bystander immune cell migration across the barrier. We further review evidence from in vitro and in vivo models that illustrate both protective and deleterious roles of CTLs at the neurovascular interface. By clearly specifying these mechanisms and their disease‑specific contexts, this review establishes a unified framework for future investigations aimed at preserving BBB function while maintaining effective CTL‑mediated immunity.

**Supplementary Information:**

The online version contains supplementary material available at 10.1186/s12967-025-07288-3.

## Introduction

The blood-brain barrier (BBB) is a protective membrane that shields the central nervous system (CNS) from blood-borne toxins and pathogens, thereby preserving CNS homeostasis [1]. BBB dysfunction is a common pathological feature in many neurological diseases. Compromised BBB integrity or impaired function can significantly contribute to the progression of these conditions. In numerous neurological disorders, BBB disruption is frequently accompanied by an immune response within the nervous system. This response includes innate immunity, primarily neuroinflammation [2, 3], and adaptive immunity involving T and B cells [4–6]. Current research focuses primarily on T cell immunity in adaptive responses, as both innate and adaptive responses are essential for maintaining BBB function.

The innate immune system rapidly and nonspecifically responds to foreign pathogens or damaged cells by recognizing pathogen-associated molecular patterns (PAMPs) or damage-associated molecular patterns (DAMPs) [7]. In contrast, the adaptive immune system is activated over a longer period, involving the precise activation of T lymphocytes and B lymphocytes that are highly specific for their targets [8]. In general, B lymphocyte immune function is primarily mediated by antibodies secreted by their differentiated plasma cells following interaction with soluble antigens binding to the B cell receptor (BCR) [9]. T cell immunity operates through cell-to-cell interactions when the T cell antigen receptor (TCR) complex encounters peptide antigens presented by antigen-presenting cells (APCs). APCs present antigens via major histocompatibility complex class I or II (MHC I and MHC II), interacting respectively with the main subsets of T cells, CD8-positive (CD8^+^) and CD4-positive (CD4^+^) T cells [10].

This review examines cytotoxic T lymphocytes (CTLs) as an example of how T lymphocyte-mediated acquired immunity regulates to BBB dysfunction and its mechanisms. Unlike other reviews that predominantly focus on the role of innate immunity, such as neuroinflammation, in BBB function, this study concentrates on CTLs, explaining their targeting mechanisms, actions, and involvement in BBB dysfunction in neurological disorders. Therefore, the findings of this paper enhance the foundational knowledge of T lymphocyte immunity and BBB-related research, and suggest future research directions.

## BBB structure and basic function

The BBB serves as a regulated interface between the peripheral circulation and the central nervous system (CNS) [11]. Although its existence was first noted in 1885, the precise nature of the BBB remained a topic of debate well into the 20th century [12]. The detailed process of discovering and naming the BBB is summarized in Supplementary Tables 1 and briefly described as follows: in 1885, Paul Ehrlich reported that the brain is isolated from the bloodstream [13]. Subsequently, Edwin Goldman, Ehrlich’s student, demonstrated that when Evans blue dye was injected into the ventricles, only the brain and spinal cord were stained, while peripheral organs remained unstained [14]. In 1922, Lina Stern introduced the term “barrière hémato-encéphalique” in French, which was later translated to “blood-brain barrier” [15]. The BBB is a multicellular vascular structure composed of brain microvessel endothelial cells, pericytes, astrocytes, neurons, and microglial cells. Junctional complexes, including tight and adherens junctions, are present at intercellular junctions within the BBB and are crucial for maintaining its low permeability [16]. A brief summary of the main functions of these components is given in Supplementary Table 2. The BBB forms a physical and metabolic barrier that separates the CNS from peripheral tissues, protecting the brain by maintaining a stable environment [17, 18]. However, it also restricts drug entry into the CNS, complicating the treatment of brain diseases such as neurodegenerative disorders and brain cancer [19, 20]. Numerous studies have elucidated the BBB’s physiological functions, including brain protection. In addition to serving as a physical and metabolic barrier against harmful substances, the BBB maintains CNS homeostasis, facilitates the selective transport of nutrients, ions, and signaling molecules, and modulates neuroinflammatory response [21–23]. Wu et al. (2023) have detailed the functions of the BBB and the role of each component in their comprehensive review [11].

## CD8^+^ CTLs

T lymphocytes are divided into two distinct functional subgroups: CD4^+^ T lymphocytes and CD8^+^ T lymphocytes. CD4^+^ T cells are known as T helper cells (Th), whereas CD8^+^ T cells are referred to as CTLs [24]. Generally, CTLs act as powerful defenders against viral infections or intracellular pathogens by regulating the secretion of perforin and proteases in target cells, which induce apoptosis [25]. CD4^+^ T cells indirectly contribute to infection clearance by modulating the activity of other immune cells, such as macrophages, neutrophils, B cells, and CD8^+^ T cells [24]. However, pre-clinical and clinical studies have demonstrated that CD4^+^ T cells possess cytotoxic programs and can directly kill cancer cells. Additionally, the cytotoxic function of CD4^+^ T cells has been observed in other diseases, such as infections and autoimmune disorders [26–28]. In this section, we primarily discuss the production and activation of CTLs, as well as the mechanisms by which CTLs kill target cells.

Although CD8^+^ and CD4^+^ T lymphocytes represent the principal effector subsets highlighted in this review, emerging evidence underscores the essential contribution of additional T cell subsets, notably regulatory T cells (Tregs) characterized by the CD4^+^CD25^+^FOXP3^+^ phenotype [29, 30]. These cells are instrumental in preserving immune homeostasis and curbing excessive neuroinflammation [29]. By suppressing autoreactive T cell activity, Tregs facilitate peripheral immune tolerance [31, 32] and may secondarily modulate the structural and functional integrity of the BBB.

### The differentiation of T cells

**Fig. 1:**
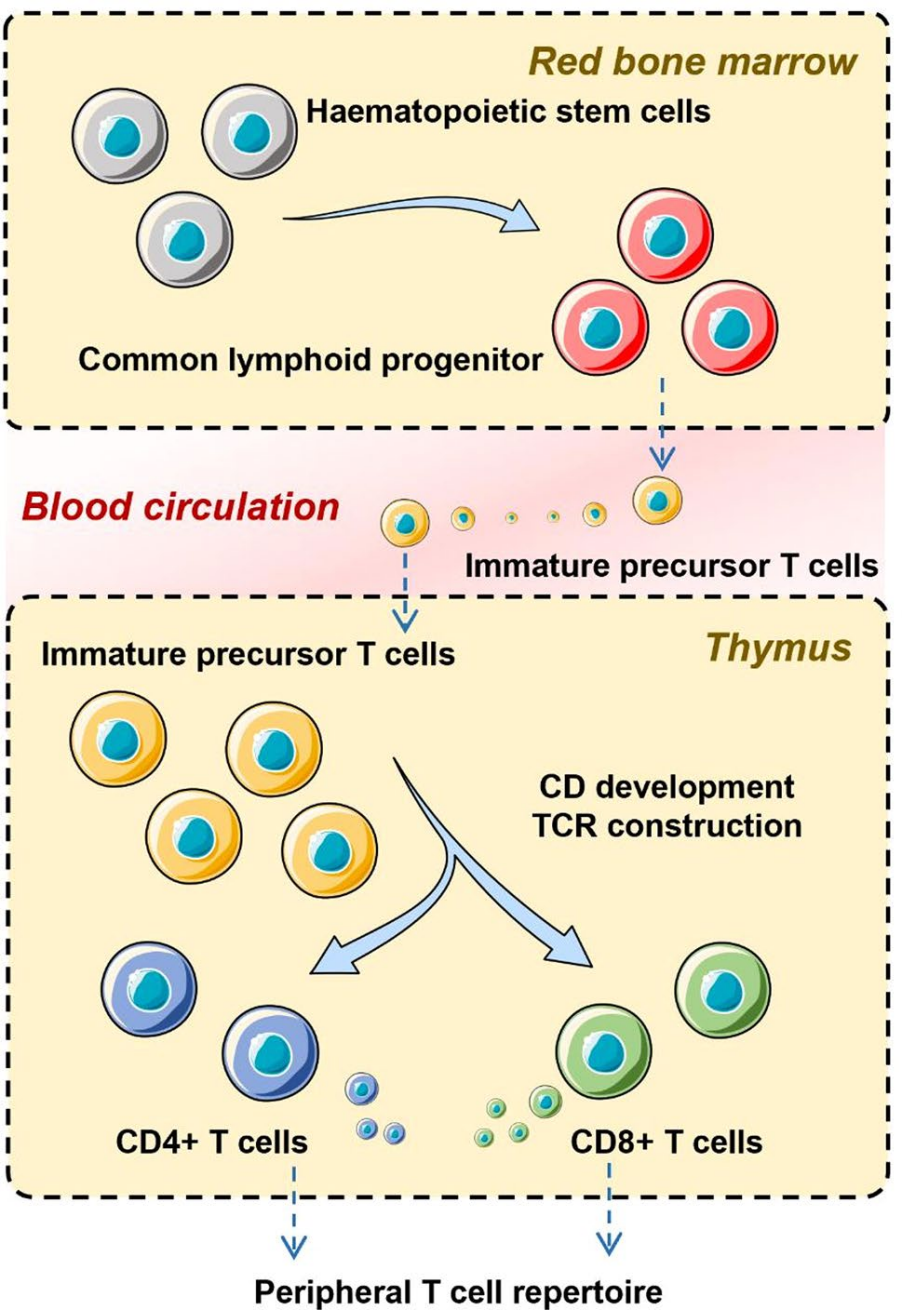
Schematic representation of the differentiation of T cells from common lymphoid progenitors. Schematic representation of the differentiation of T cells from common lymphoid progenitors. Common lymphoid progenitor (CLP) cells, which originate in the red bone marrow, give rise to immature precursor T cells. These precursor cells are initially double-negative for both TCR and CD proteins. Thymic chemotactic factors, such as thymotaxin, thymosin, and thymopoietin, guide these double-negative precursor T cells from the bloodstream into the thymus. Within the thymus, thymic cells present MHC I and II molecules to the developing T cells, prompting the expression of TCR and CD proteins. This interaction ensures positive selection, which leads to the survival of T cells that can bind MHC molecules with at least weak affinity. T cells that recognize MHC I differentiate into CD8^+^ T cells, while those recognizing MHC II develop into CD4^+^ T cells. Furthermore, CD4^+^ T cells may differentiate into specialized subsets such as Th cells or Treg cells, depending on the presence of specific cytokines and stromal signals. Abbreviations: CLP, common lymphoid progenitor; TCR, T-cell receptor; MHC, major histocompatibility complex; CD, cluster of differentiation; Th, T-helper; Treg, T-regulatory

CTLs differentiation occurs in three distinct stages based on their sites of action. The first stage takes place in the red bone marrow, where common lymphoid progenitor cells differentiate into immature precursor T cells. Due to their high migratory capacity, these precursor T cells enter the circulatory system. Chemotactic agents or thymic factors from the thymus (such as thymotaxin, thymosin, and thymopoietin) direct their migration to the thymus, marking the second stage (circulatory system) and the third stage (thymus) of differentiation. In the thymus, the essential differentiation process involves thymic cells presenting CD- and TCR-positive T cells to MHC I and MHC II molecules to evaluate T-cell reactivity and direct their maturation pathways. T cells with TCR affinity for MHC I become CD8^+^ T cells, whereas those with TCR affinity for MHC II become CD4^+^ T cells [24]. Depending on cytokine and stromal cell signaling, they may further differentiate into T-helper and T-regulatory cells, both of which are subsets of CD4^+^ T cells [33, 34]. The aforementioned process is illustrated in Fig. 1.

Tregs, characterized by high expression of CD25 and the transcription factor FOXP3 on CD4⁺ T cells, are indispensable for maintaining peripheral immune tolerance and suppressing autoimmunity [29, 30]. They exert immunosuppressive effects through both direct cell–cell interactions and the secretion of anti-inflammatory cytokines, including interleukin-10 (IL-10) and transforming growth factor-beta (TGF-β) [35]. In addition to Tregs, other T cell subsets, such as T helper 17 (Th17) cells and γδ T cells, also participate in the regulation of neuroinflammation via distinct cytokine signatures and differential tissue-homing capacities [36, 37]. Increasing evidence indicates that Tregs contribute to the preservation of BBB integrity by attenuating proinflammatory cytokine production and promoting the stabilization of endothelial tight junctions in CNS autoimmune disease models [38, 39].

### The activation of CD8^+^ CTLs

The activation of CD8^+^ CTLs is initiated through their initial interactions with target cells. Three critical components in this process are APCs, as well as the TCR and CD28 on CTLs.

APCs are essential in mediating interactions between T cells and their targets. Initially, APCs bind to target substances such as cancer cells, pathogens, viruses and others. Through phagocytosis and the action of proteases, these targets are degraded into antigenic peptide fragments, forming the MHC I -APC-target complex. CD8^+^ T cells recognize the MHC I antigen peptide complex on this structure. Upon contact, T cells adhere to the complex and scan its surface. By homing towards chemokine and integrin gradients on APCs or target cells, CD8^+^ T cells form immunological synapses between their supramolecular activation complex and adhesion molecules, such as intercellular adhesion molecules, on the target cell surface [40, 41].

**Fig. 2:**
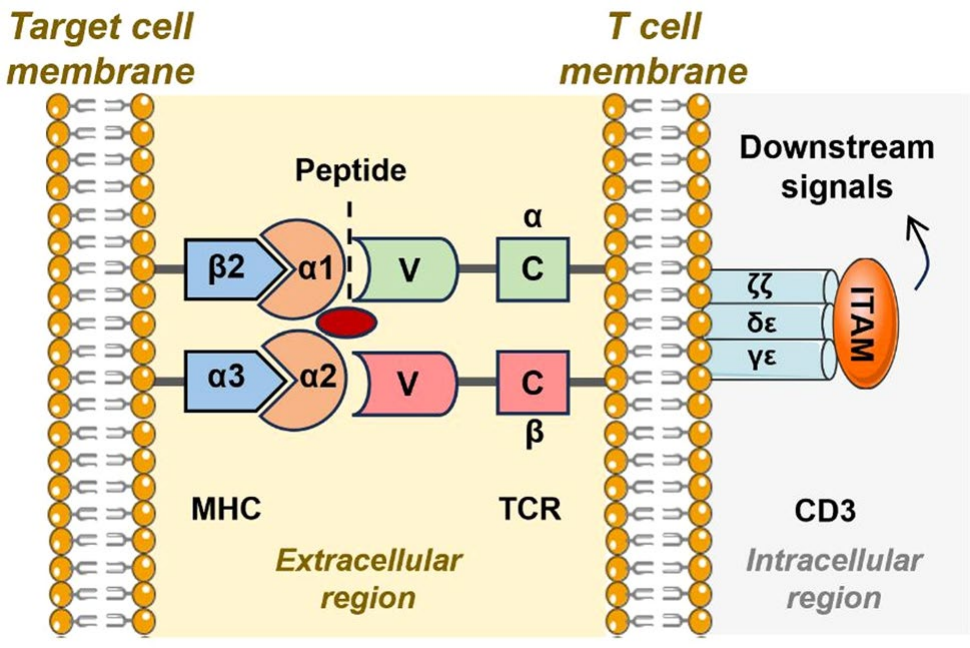
Schematic representation of T cell activation upon recognition of antigenic peptides. The variable (V) regions of the α and β chains of the TCR specifically recognize and bind to antigenic peptides presented by MHC I molecules on target cells. This interaction is enhanced by the co-receptor CD8, which binds to both the TCR and MHC I, stabilizing the TCR–CD3 complex at the MHC–peptide interface. This stable interaction leads to the phosphorylation of ITAMs within the CD3 subunit of the TCR complex. The phosphorylation of ITAMs activates downstream signaling cascades that result in the activation of transcription factors such as NF-κB, NFAT, and AP-1, ultimately driving the proliferation and effector function of the CD8^+^ T cell. These effector functions include cytokine secretion and the generation of cytotoxic molecules such as perforin and Granzyme B. Abbreviations: TCR, T-cell receptor; MHCI, major histocompatibility complex class I; CD, cluster of differentiation; ITAM, immunoreceptor tyrosine-based activation motif; NF-κB, nuclear factor kappa-light-chain-enhancer of activated B cells; NFAT, nuclear factor of activated T-cells; AP-1, activator protein 1

During immunological synapse formation, TCR and CD28 on CD8^+^ T cells play critical roles. The TCR is a complex structure composed of the antigen-binding subunit (TCRαβ) non-covalently linked with three CD3 co-receptor signaling subunits (ζζ, CD3δε, and CD3γε) [42]. The intracellular CD3 contains immunoreceptor tyrosine-based activation motifs (ITAMs), which are essential for linking intracellular tyrosine kinase functions [42]. Hence, the CD3-ITAM pathway in TCR is crucial for assembling and transmitting intracellular signals following surface recognition by TCR. After TCR is activated by the MHC I -APC-target complex, a separate co-stimulatory signal is required; otherwise, T cells will not fully activate, leading to inactivity or apoptosis. This additional signal comes from the CD28 receptor on CD8^+^ T cells, which binds to CD80/B7.1 or CD86/B7.2 on APCs, promoting T cell proliferation and cytokine production, such as IL-2 [43]. The aforementioned process is illustrated in Fig. 2. During this process, CD28 induces multiple signaling pathways in T cells, such as the PI3K-AKT and NF-κB pathways, leading to increased Bcl-xL expression and enhanced T cell survival [44]. Additionally, CD28 signaling protects CD8^+^ T cells from reacting to self-antigens, thereby reducing the risk of tissue damage and autoimmunity. A more detailed description of CD8^+^ T cell activation can be found in the review published by Hans Raskov in 2021 [45].

### The CD8^+^ CTLs-mediated mechanism of target-cell death

Once activated, CD8^+^ CTLs demonstrate their potent cytotoxic abilities. As reported in various studies, CD8^+^ CTLs bind to the Fas receptor on the target cell via the Fas ligand (FASL) on their surface, activating the death domain within the target cell. This activation subsequently triggers caspases and nucleases, leading to the fragmentation of the target cell’s DNA [46]. More importantly, the cytotoxic activity of CD8^+^ CTLs primarily depends on the release of granules containing granzymes, perforin, cathepsin C, granulysin, and other effector molecules. These granules fuse with the target cell membrane, allowing the effector molecules to enter the target cell and create pores in the endosomal membrane, resulting in cell destruction [47, 48]. These processes occur within the immunological synapse (IS) formed between the CD8^+^ CTLs and the target cell [41]. In brief, CD8^+^ T cells exhibit persistent motility when interacting with target cells, which facilitates pore formation in the target cell membrane [47]. This allows the release of cytotoxic granules containing granzymes, perforin, cathepsin C, and granulysin, which fuse with the target cell membrane to initiate cell death [47]. Alternatively, the target cell may internalize a complex of granulysin, perforin, and granzymes through endocytosis of the cytotoxic T-cell membrane [48]. Once internalized, perforin and granulysin create pores in the endosomal membrane, allowing granzymes to escape into the cytoplasm, where they trigger apoptosis [48].

The IS is the interface where CD8^+^ CTLs engage with target cells, facilitating TCR-mediated signaling and secretory events. Similar to natural killer cells, the initiation of IS formation in CTLs involves two signals [49]: the absence of MHC I recognition (disinhibition) and a positive signal from germline-encoded activation receptors that bind to specific ligands on target cells, such as lectins or hemagglutinins. Once antigenic peptides are recognized by the TCR on CTLs, the IS is formed, triggering complex signaling cascades involving the TCR, CD28, and associated pathways. These cascades lead to the realignment of the Golgi complex and microtubule network, with the microtubule-organizing center repositioning towards the IS and microtubules extending towards the distal pole. Along these microtubule tracks, effector granules are transported to the IS for secretion [50]. The mechanism by which granules enter target cells is complex and involves multiple modifications to the target cell’s plasma membrane. A critical factor in this process is the accumulation of Orai Ca2^+^ channels and the involvement of t-SNARE syntaxin11. The activation of Orai Ca2^+^ channels occurs in conjunction with IP3/Ca2^+^-dependent activation and the translocation of STIM proteins to the endoplasmic reticulum near the IS. These activated STIM proteins interact with Orai channels, forming the store-operated Ca2^+^ release-activated Ca2^+^ complex, which drives store-operated Ca2^+^ entry [51–53]. The increase in cytosolic Ca2^+^ concentration is further enhanced by adjacent mitochondria [54, 55], ensuring optimal synaptic activation [56, 57]. Concurrently, t-SNARE syntaxin11, essential for lysosomal granule fusion, relocates to the IS and integrates into the plasma membrane through a VAMP8-dependent mechanism [58, 59]. This coordination ensures the precise positioning of release machinery components. Additionally, further modifications to the target cell membrane involve interactions between proteins on the granules and the target membrane, such as Rab27/Munc13 and VAMP/Munc18. Although the specific details of these molecular mechanisms are extensively covered in various reviews [60], they are not elaborated on here. These interactions highlight the intricate regulation of granule fusion and release, which is crucial for the effective cytotoxic response of CTLs.

An overactivated CD8^+^ CTLs response can be detrimental, leading to autoimmune disorders, rejection of transplanted cells, and graft-versus-host disease. This is because the lytic machinery of CTLs can mistakenly target self-tissues or host tissues [61]. To prevent such uncontrolled activation, immune checkpoint molecules, which are transiently expressed inhibitory receptors on the cell surface, are essential. They regulate CD8^+^ CTLs activation, ensuring the immune response is properly modulated even in the presence of strong activation signals [62]. This checkpoint molecule is also present in other immune cells, including natural killer cells and activated macrophages, where they perform similar regulatory functions. Key checkpoint molecules include programmed cell death receptor 1 (PD-1 or CD279), CTLA-4, lymphocyte-activation gene 3 (LAG-3), T-cell immunoglobulin and mucin domain-3 (TIM-3), T-cell immunoreceptor with Ig and ITIM domains (TIGIT), and inducible T-cell co-stimulatory receptor (ICOS). The mechanisms by which these immune checkpoints function have been extensively reviewed [63, 64], and in this paper, their main modes of action are displayed in Supplementary Table 3. However, malignant tumor cells can exploit these inhibitory signals to evade the immune response and enhance their own survival [65].

The development of monoclonal antibodies targeting immune-inhibitory receptors, known as checkpoint inhibitors, represents a major breakthrough in immuno-oncology, significantly improving the clinical outcomes of various cancers [66]. This therapeutic approach enhances antitumor immune responses while also revitalizing exhausted CD8^+^ T cells, thereby increasing tumor cell eradication. Among these therapies, anti-PD-1 agents have been particularly transformative in the treatment of metastatic melanoma, demonstrating remarkable clinical efficacy [67, 68]. Several checkpoint inhibitors targeting the PD-1 pathway have received approval in the United States, including three PD-1 inhibitors (pembrolizumab, nivolumab, and cemiplimab), and three PD-L1 inhibitors (atezolizumab, avelumab, and durvalumab). Current research focuses on improving the efficacy and reducing the toxicity of these agents by combining them with other therapeutic modalities, such as immunotherapies or cytotoxic chemotherapies. Notably, the combination of PD-1/PD-L1 inhibitors with CTLA-4 inhibitors has yielded promising clinical outcomes, as demonstrated by the approval of nivolumab in combination with ipilimumab for the treatment of metastatic melanoma, advanced renal cell carcinoma, and mismatch repair-deficient colorectal cancer [69, 70].

## CD4^+^ CTLs

### Ontogeny and differentiation of CD4⁺ CTLs

CD4⁺ CTLs differentiate from naive CD4⁺ T cells under conditions of persistent antigen stimulation and pro-inflammatory cytokines such as IL-2, IL-15 and IL-22 [71–73]. Transcription factors T-bet and Eomesodermin coordinate the acquisition of cytotoxic programs by upregulating perforin and granzyme B expression [73, 74]. Co-stimulatory signals via CD28 and 4-1BB further enhance CD4⁺ CTL expansion and survival [75]. In chronic infections, such as tuberculosis, CD4⁺ CTLs increase in frequency and partially restore pathogen clearance when CD8⁺ CTLs exhibit an exhausted phenotype marked by PD-1 and TIM-3 upregulation [76, 77]. Similarly, in autoimmunity models, CD4⁺ CTLs compensate for impaired CD8⁺ responses by targeting MHC II-expressing antigen-presenting cells and sustaining local cytotoxicity [78].

### Effector mechanisms of CD4⁺ CTLs

Conventional CD4^+^ T cells, including thymus-derived FOXP3 regulatory T cells, are part of the Th cell lineage, characterized by a TCR that recognizes MHC II [79]. The functional diversity of Th subsets is further expanded by the presence of CD4^+^ T cells with cytotoxic capabilities, known as CD4^+^ CTLs. Initially, these CD4^+^ CTLs were dismissed as artifacts from exhausted, long-term cultured T cell lines or miscategorized within the Th1 subset [80, 81]. However, research over the past decades has demonstrated that CD4^+^ CTLs are a distinct Th subset with antigen-specific cytotoxic activity, observable in both humans and mice [82, 83].

CD4^+^ CTLs, similar to CD8^+^ T cells, utilize two primary effector mechanisms to eliminate target cells [84, 85]. The first involves the release of cytotoxic granules containing perforin and granzyme B, which induce perforin oligomerization and pore formation in the target cell membrane [86]. The second mechanism involves Fas/FasL-mediated apoptosis, where FasL on CD4^+^ CTLs binds to Fas receptors on target cells, activating Caspase 8 and subsequently Caspase 3, leading to apoptosis. Detailed descriptions of these mechanisms are provided in the “CD8^+^ CTLs” section of this paper. In contrast to CD8^+^ T cells, which recognize antigens presented by MHC I molecules, CD4^+^ CTLs recognize peptides presented by MHC II molecules on APCs. Therefore, it is unlikely that CD4^+^ CTLs simply substitute the function of CD8^+^ CTLs.

### Compensatory roles in chronic infection and autoimmunity

The distinctive characteristic of CD4^+^ CTLs is their capacity to kill target cells, mirroring and complementing the cytotoxic function of CD8^+^ T cells. Although CD4^+^ CTLs are found in low numbers under normal conditions [86], their population increases significantly during chronic viral infections such as those caused by cytomegalovirus, dengue virus, ectromelia virus, lymphocytic choriomeningitis virus, and other pathogens [87–90]. Growing evidence suggests that the cytotoxic activities of CD4^+^ T cells against infected or transformed cells likely compensate for the reduced killing efficacy of exhausted CD8^+^ CTLs, which can be inhibited by virus-induced checkpoint molecules [91]. For instance, during chronic Mycobacterium tuberculosis (Mtb) infection, T-cell immunity is suboptimal due to the expression of inhibitory receptors like PD-1 and TIM-3, resulting in reduced cytokine production [76, 77]. Consequently, CD8^+^ T cells exhibit an exhausted phenotype, and CD4^+^ T cells adopt a cytotoxic profile marked by the expression of Tbx21, potentially compensating for the impaired function of CD8^+^ T cells during active tuberculosis [92].

## The role of CTLs in the regulation of BBB function

The association between the BBB and CTLs was first reported by Wyde et al. in 1983 [93], as recorded in the PubMed database. Wyde and colleagues compared the dissemination of a neurovirulent strain of influenza A/WSN (HON1) virus from infected lungs to brains of thymus-deficient nude and immunocompetent furred mice, both inoculated intranasally. Their results revealed that, in immunocompetent mice, the virus was typically cleared from the lungs of survivors, with minimal cases of viral spread to the brain. In contrast, nude mice exhibited frequent and early deaths, with significant viral titers in the brain and histological evidence of encephalitis. Notably, adoptive immunization of nude mice with CTLs, which had been stimulated in vitro 24 h after intranasal challenge, led to a reduction in both brain virus titers and mortality [93]. These findings underscored the crucial role of T lymphocytes in inhibiting the dissemination of neurotropic viruses from the lungs to the brain.

Wyde’s pioneering study suggested for the first time that T lymphocytes are integral to the BBB’s defense against viral invasion. In the 1980s, Hafler and colleagues further examined and reviewed the role of T cells in multiple sclerosis and other inflammatory central nervous system diseases [94]. For instance, Hafler et al. initiated clinical trials using anti-T-cell murine monoclonal antibodies (MAbs) to treat multiple sclerosis, aiming to develop a targeted and non-toxic immunotherapy [95]. During infusions with anti-T11, a pan-T-cell monoclonal antibody targeting the CD2 receptor, they observed that the antibody bound to peripheral blood T cells without inducing significant cell lysis, and did not immediately modulate the CD2 surface structure. Additionally, they found that the BBB remained relatively impermeable to the antibody. This unique scenario allowed researchers to study the migration of peripheral T cells into the CNS in patients with progressive multiple sclerosis.

Following these groundbreaking studies, researchers began investigating how CTLs contribute to neurological dysfunction, particularly by crossing or disrupting the BBB. In this context, we focus on the role of CTLs in maintaining the integrity of the BBB and their associated functions in neurological conditions, particularly brain tumors, non-tumor neurological diseases such as multiple sclerosis and Parkinson’s disease, as well as virus-induced or pathogen-induced neurological disorders.

### Brain-related tumors

#### Brain metastases of tumors

The association between CTLs and BBB in brain tumor models was initially reported by Gordon et al. using a P511 mastocytoma cell tumor model [96]. Their research demonstrated that, on the seventh day following cannula implantation in the cerebral cortex, brain tumors developed while the BBB remained intact. Importantly, the population of P511-specific non-cytolytic CTL precursors (pCTLs) were identified at the brain tumor site, suggesting that these pCTLs, generated in the periphery, migrated to the brain tumor area. The incomplete activation of these cells, likely due to the inhibitory microenvironment of the central nervous system, indicated that the unique structure of the BBB prevents their full activation, thus reducing their cytotoxic potential. Furthermore, when the tumor cells were injected at a flank site, similar phenomena were observed in the brain metastasis model of P511 mastocytoma cells [96].

#### Glioma

Glioblastoma multiforme (GBM) is the most common and aggressive malignant primary brain tumor in adults. Focused ultrasound (FUS) can temporally and locally open the BBB. In a GBM mouse model, Chen et al. utilized FUS to disrupt the BBB, leading to significant changes in tumor-infiltrating lymphocyte (TIL) populations within the brain, particularly increasing the number of CD3^+^CD8^+^ CTLs in the tumor region. This resulted in notable inhibition of tumor progression and improved survival rates in the animals [97]. Oncolytic virotherapy is another promising approach to improve the poor prognosis of malignant brain tumors. The rat H-1 parvovirus (H-1PV) has shown tumor suppression in preclinical glioma models through direct oncolysis and stimulation of anti-cancer immune responses [98, 99]. Because the virus can penetrate the blood-brain/tumor barrier and spread extensively within the tumor, significant changes were observed in the tumor microenvironment upon viral infection. These changes included microglia/macrophage activation and CTLs infiltration, indicating that H-1PV may trigger an immunogenic response [98, 99]. Numerous similar studies have reported other methods and vectors capable of altering the brain’s immune microenvironment, such as the RNA-modification of T Cells, modified nanoparticles, and others [100–104]. These approaches must successfully penetrate the BBB—a major challenge in brain cancer treatment—and increase CTLs infiltration at the tumor site. Notably, the increased CTLs are predominantly CD8 positive [100–104]. Thus, current research on brain tumors, CTLs, and the BBB primarily seeks methods to cross the BBB and enhance the cytotoxic function of immune cells, such as CD8^+^ CTLs, at the tumor site. However, there is no research on the direct effects of CTLs on the BBB in brain tumors.

### Non-neoplastic neurological diseases or dysfunctions

#### Multiple sclerosis (MS)

MS is a central nervous system disease characterized by inflammation and autoimmunity. In 1993, researchers discovered that peripheral T cells from patients with acute MS exhibit a cytotoxic effect on brain endothelial cells [105]. This observation indicates that T cell-induced cytotoxicity towards brain endothelial cells might play a role in increasing BBB permeability and triggering immune responses in acute MS [105].

The Theiler’s murine encephalomyelitis virus (TMEV) model is a key tool for studying MS. Researchers have used this model to explore the role of CTLs in MS, with significant contributions from Georgette L. Suidan’s team between 2008 and 2012 [106–108]. They found that CD8^+^ CTLs might disrupt the BBB through mechanisms involving perforin and vascular endothelial growth factor (VEGF). Their research suggested that, unlike their typical cytotoxic role against harmful cells, CD8^+^ CTLs use a non-apoptotic perforin-dependent mechanism to break down BBB tight junctions. This mechanism involves the activation of astrocytes, alteration of BBB tight junction proteins, and increased CNS vascular permeability [106]. Another pathway includes VEGF, where CD8^+^ CTLs interact with neurons, either directly or indirectly through other immune cells, leading to VEGF upregulation, which disrupts tight junctions and increases vascular permeability [107, 108].

Researchers have also studied the relationship between CTLs and the BBB in MS, particularly focusing on the ability of CTLs to penetrate the BBB. Studies have shown that in MS, B cell-derived interleukin-15 (IL-15) increases the proportion of CD8^+^ CTLs in the brain and enhances their ability to cross the BBB. However, the molecular mechanisms by which IL-15 facilitates CD8^+^ CTLs migration across the BBB remain unclear [109]. Other researchers hypothesize that this process may involve microRNAs of CTLs or P-glycoprotein in brain endothelial cells [110]. Aya A. Elkhodiry found a significant correlation between the downregulation of microRNA-155 in CD8^+^ CTLs isolated from MS patients’ blood samples and the upregulation of intracellular adhesion molecule 1 (ICAM1) and integrin subunit beta 2 (ITGB2), both of which are critical for migration through the BBB [110]. Similarly, Gijs Kooij’s 2014 study demonstrated that endothelial P-glycoprotein mediates the migration of CD8^+^ CTLs across the BBB [111]. Their research showed that reducing P-glycoprotein expression in endothelial cells using shRNA significantly decreased the transendothelial migration and adhesion capabilities of CD8^+^ and CD4^+^ CTLs in an in vitro BBB model. This finding was further corroborated in vivo using cell-specific CCL2 knockout mice, revealing that P-glycoprotein regulates CD8^+^ T cell migration via CCL2 secretion [111].

Additionally, CD4^+^ CTLs have been reported to play a crucial role in MS. These CD4^+^ T cells co-express NKG2D, an activating receptor predominantly expressed on NK cells, CD8^+^ T cells, and γδ T cells in humans and mice [112]. Tobias Ruck et al. reported that these CD4^+^ NKG2D^+^ T cells exhibit high levels of migration, activation, and cytolytic activity. In an in vitro BBB model, NKG2D facilitated the migration of CD4^+^ NKG2D^+^ cells through endothelial cells [113].

#### Parkinson’s disease

In Parkinson’s disease (PD), a progressive neurodegenerative disorder affecting 2–3% of the population over 65 years old [114], peripheral CD4^+^ CTLs have been also reported to regulate BBB dysfunction. In 2023, Shi et al. used single-cell RNA sequencing to elucidate the potential mechanisms by which CD4^+^ T cells contribute to BBB disruption [115]. Their study revealed a significant increase in the proportion of PD-related CD4^+^ CTLs in the peripheral blood mononuclear cells of PD patients. Moreover, these CD4^+^ CTLs exhibited significantly elevated expression of the *Ifng* gene, which is particularly sensitive to endothelial cells compared to other midbrain cell types. Further cell-cell communication analysis identified that during the process of CD4^+^ CTLs weakening endothelial cell tight junctions, IFNG/IFNGR1 and SPP1/ITGB1 were the primary signaling pathways between CTLs and endothelial cells [115].

#### Epilepsy

In epilepsy research, direct evidence of CTLs regulating BBB function is currently lacking, but several studies have explored related functional aspects. Nicola Marchi and colleagues conducted a study using splenectomy to immunosuppress rats, which reduced various immune cells, including CTLs, and subsequently decreased mortality in a pilocarpine-induced rat epilepsy model [116]. Furthermore, they induced epilepsy in perforin-deficient mice with pilocarpine and observed reduced BBB damage compared to controls [116]. Since perforin is a key effector molecule for CTL-mediated cytotoxicity, this study indirectly supports the idea that CTL-perforin pathways contribute to BBB damage [116], similar to findings by Suidan’s team in the TMEV model [117]. Another study examined the effects of rapamycin (RAP) on CTLs and BBB in epilepsy [118]. This research reported that RAP increased the levels of total T cells (CD3^+^/CD45^+^) and T helper cells (CD3^+^/CD4^+^) in epileptic rats while reducing the levels of CTLs (CD3^+^/CD8^+^). Simultaneously, harmful BBB factors such as MMP-9, MMP-2, and inflammatory cytokines were decreased [118]. This study highlighted an inverse relationship between BBB function and CTLs in an epilepsy model but did not further analyze the underlying mechanisms or provide detailed correlations.

#### Hemorrhagic stroke

In hemorrhagic stroke, CCL5 in astrocytes has been shown to play a critical role in the interaction between peripheral CTLs and astrocytes, leading to BBB disruption. Zhou et al. identified CCL5 as one of the top upregulated genes in RNA sequencing results from astrocytes activated by IL-1α, TNF-α, and complement component 1q treatment [119]. Functional validation demonstrated that knocking out CCL5 in astrocytes reduced CD8^+^ T cell infiltration into the brain, but did not affect the infiltration of CD4^+^ T cells and myeloid cells. Moreover, reduced CCL5 expression decreased BBB disruption following hemorrhagic stroke, although this protective effect was nullified by the supplementation of CD8^+^ CTLs [119].

#### Susac syndrome

Susac syndrome (SuS) is a rare neuroinflammatory disease characterized by endothelial dysfunction in the central nervous system, manifesting as focal microangiopathy that affects the small-to-medium-sized vessels of the brain, retina, and inner ear [120, 121]. The pathogenesis of SuS remains highly controversial, with the most widely accepted theory suggesting an autoimmune process [122]. In a 2019 publication, Catharina C. Gross and colleagues proposed that SuS is an endothelial injury disease driven by CTLs targeting an unknown antigen [123]. Specifically, an unidentified antigen activates CD8^+^ CTLs, enabling them to secrete granzyme B and perforin. These activated CTLs then accumulate in the microvasculature of the brain, retina, and inner ear, adhere to endothelial cells, and induce apoptosis via granzyme B and perforin, thereby disrupting the BBB and causing localized microhemorrhages. This initiates a cascade of neuroinflammation, leading to the loss of astrocytes, oligodendrocytes, neurons, and axons. Eventually, ischemic lesions infiltrate surrounding astrocytes, transforming into gliosis [123]. Throughout the disease progression, the granzyme B and perforin-dependent damage by CD8^+^ CTLs to endothelial cells and the BBB is a critical process. Understanding the activation mechanisms of CD8^+^ CTLs is crucial for advancing the treatment and prevention of Susac syndrome.

In 2023, Carmen Gonzalez-Fierro further validated Gross’s hypothesis using an in vitro co-culture model of primary brain microvascular endothelial cells and CD8^+^ CTLs [124]. This study confirmed that perforin-dependent cytotoxicity is a key mediator of endothelial cell death, suggesting this mechanism as a foundational aspect of SuS pathogenesis [124].

#### Schizophrenia

N. Müller examined the expression of adhesion molecule receptors, specifically VLA-4 and LFA-1, on Th (CD4^+^) and T suppressor/cytotoxic (CD8^+^) lymphocytes in patients with schizophrenia, both before and during antipsychotic treatment [125]. The investigation revealed that the proportion of VLA-4^+^/CD4^+^ and VLA-4^+^/CD8^+^ cells increased significantly during antipsychotic therapy. Furthermore, VLA-4^+^/CD4^+^ and LFA-1^+^/CD4^+^ cells were strongly linked to disturbances in the BBB [125]. Since this study was conducted in the late 20th century, the researchers did not validate these correlations or delve into the underlying mechanisms comprehensively.

### Virus-induced or pathogen-induced neurological disorders

#### Cerebral malaria

Cerebral malaria, a severe complication of Plasmodium falciparum infection, involves associations between CTLs and BBB similar to those seen in neurological diseases like SuS and MS [106, 123]. In cerebral malaria, CD8^+^ T lymphocytes induce endothelial cell apoptosis through a perforin-dependent mechanism, contributing to the observed lethality in murine models [126, 127]. Researchers have explored strategies to mitigate CTLs toxicity to the BBB in experimental malaria, such as modulating the functions of antigen-presenting cells and controlling the migration of activated T cells [128–131]. Johanna F. Scheunemann has comprehensively reviewed these findings [132]; thus, further elaboration is unnecessary here.

#### Human T-cell leukaemia virus 1

Human T-cell leukemia virus type 1 (HTLV-1) infection can lead to T-cell leukemia and inflammatory diseases, most notably HTLV-1-associated myelopathy/tropical spastic paraparesis (HAM/TSP) [133]. In TSP/HAM, HTLV-1-infected T cells, anti-HTLV-1 cytotoxic T cells, and macrophages infiltrate the cerebrospinal fluid, indicating that the disease involves disruption of the blood-brain barrier (BBB) [134]. Nirit Mor-Vaknin, in 1998, demonstrated that HTLV-1-infected T cells can fuse with and damage astrocytes in vitro, proposing that the destruction of astrocytes by HTLV-1-infected T cells leads to BBB disruption [134]. Furthermore, research by Guangyong Ma has shown that peripheral HTLV-1-infected T cells can transfer HTLV-1 to brain endothelial cells, causing BBB damage [135]. Thus, peripheral T-cell-mediated viral transmission may be a key mechanism in HTLV-1-induced BBB disruption.

#### Dengue virus

In acute viral encephalitis induced by Dengue virus (DENV) infection, CD8^+^ CTLs likely play a major role. Tsung-Ting Tsai and colleagues found that in DENV-infected mice [136], CD8^+^ CTLs infiltration into the central nervous system resulted in CNS inflammation and BBB disruption. During this process, microglial cells exhibited significant antigen-presenting cell functions, stimulating CTLs proliferation and activation. Conversely, depleting microglial cells eliminated DENV-induced antiviral cytokine expression and CD8^+^ CTLs infiltration, restoring BBB integrity and neurological function [136].

#### Lymphocytic choroid plexus meningitis virus

Lymphocytic choriomeningitis virus (LCMV) infection in mice causes fatal immunopathology and convulsive seizures through BBB disruption [137, 138]. LCMV-specific CTLs are crucial in this process. Jiyun V. Kim and colleagues reported that during acute viral meningitis, activated CD8^+^ CTLs not only damage the BBB through downstream effector molecules (e.g., IFN-γ receptor, TNF-α, Fas, granzyme, perforin) but also express various chemokines that recruit bone marrow mononuclear cells responsible for vascular injury [139].

#### Adeno-associated virus (AAV)

AAV, a member of the *Parvoviridae* family, is widely used in scientific research. Although intracranial microinjection of AAV is generally regarded as a safe and effective method for inducing transgene expression in the central nervous system, high doses of AAV can exhibit neurotoxicity and damage the BBB. This damage may be mediated by the infiltration of peripheral CTLs into the CNS. This hypothesis is supported by findings that neuronal loss induced by high-dose AAV injection can be alleviated by depleting infiltrating T immune cells [140].

### Advanced experimental models to elucidate CTL–BBB dynamics

Recent technological innovations have significantly enhanced our ability to dissect CTLs interactions with the BBB under near-physiological conditions. These models span high-resolution single-cell omics, intravital microscopy, and biomimetic “BBB-on-a-chip” platforms, each offering unique insights into CTLs trafficking, signaling, and barrier disruption.

#### Single-cell omics

Yan et al. applied droplet-based single-cell RNA sequencing to isolate and profile over 33,000 CD4⁺ CTLs from both peripheral blood and CNS infiltrates of Parkinson’s disease patients [115]. They discovered pronounced upregulation of IFNG and SPP1 in CTLs, accompanied by elevated IFNGR1 and ITGB1 expression in brain microvascular endothelial cells—identifying a pathogenic signaling axis that undermines tight junction integrity. Complementarily, Patil et al. performed single-cell transcriptomics on peripheral blood mononuclear cells (PBMCs) from healthy donors, delineating CD4⁺ CTL differentiation trajectories marked by sequential induction of cytolytic effectors GZMB and PRF1 [88].

#### Intravital imaging

Kim et al. and Phillip et al. utilized two-photon intravital microscopy in lymphocytic choriomeningitis virus (LCMV)–infected mice to visualize CTL behavior within intact brain microvasculature [139, 141]. Their studies reveal CTL crawling, arrest, and transendothelial migration guided by chemokine gradients (e.g., CXCL10), correlating precisely with localized BBB permeability increases.

#### Human BBB-on-a-chip models

Nair et al. engineered a microfluidic BBB model comprising human brain microvascular endothelial cells cultured against an extracellular matrix gel within 40 parallel channels [142]. Upon exposure to TNF-α and IL-1β, transendothelial electrical resistance (TEER) declined by ~ 30%, and adhesion molecule expression (ICAM-1, VCAM-1) increased. When primary human T cells were perfused under flow along a CXCL12 gradient, they faithfully recapitulated inflammation-driven extravasation observed in vivo.

By bridging reductionist and in vivo approaches, these advanced models afford unprecedented mechanistic resolution of CTL–BBB dynamics. Single-cell omics elucidate the molecular programs within individual CTLs and endothelial cells; intravital imaging captures real-time cellular behavior within the native microenvironment; and BBB-on-a-chip platforms provide scalable, human-relevant systems for high-throughput interrogation of immune cell transmigration. Collectively, these methodologies pave the way for targeted interventions that preserve barrier integrity while modulating neuroimmune crosstalk.

### Translational caveats and data gaps

While murine models have elucidated key mechanisms of CTL–BBB modulation, their direct extrapolation to human disease is constrained by several factors:

#### Species and model differences

Rodent and human brain microvascular endothelial cells differ markedly in tight junction composition (e.g., claudin-5 levels [143]) and transporter expression (P-glycoprotein, BCRP [144]), altering permeability and leukocyte trafficking.

#### Temporal dynamics

Experimental antigen challenges in mice typically unfold over hours to days, whereas human neurodegenerative and autoimmune disorders feature chronic, low-grade inflammation persisting for months to years. Such divergence may obscure the progressive BBB remodeling observed clinically.

#### Genetic homogeneity vs. diversity

Inbred mouse strains lack the genetic polymorphisms present in human populations (e.g., cytokine and chemokine receptor variants) [145] that critically shape CTL responses and barrier interactions.

#### Clinical data scarcity

Few studies have quantified CTL infiltration or BBB integrity in human CNS tissues. MRI and PET assessments of barrier leakage remain limited to small cohorts in multiple sclerosis [146] and post-COVID syndromes [147], whereas, post-mortem immunohistochemical analyses of CTLs are rare.

#### Underutilized human in vitro models

Although induced pluripotent stem cell (iPSC)–derived BBB organoids and microfluidic “BBB-on-a-chip” platforms can recapitulate shear stress and multicellular architecture [142, 148], they are not yet widely adopted for investigating CTL transmigration.

Addressing these gaps will demand integration of humanized animal models, longitudinal patient sampling, advanced in vivo imaging tools, and broader deployment of human BBB platforms to ensure that preclinical insights align with human pathophysiology.

## Therapeutic implications and future strategies

Translating mechanistic insights into effective therapies requires approaches that precisely modulate CTL activity at the BBB while preserving barrier integrity:

### Immune checkpoint blockade

Agents such as anti–PD‑1/PD‑L1 antibodies (e.g., nivolumab) can rejuvenate exhausted CTLs [149, 150] but may aggravate BBB permeability through enhanced cytokine release.

### Chemokine-axis blockade

Targeting chemokine receptors (e.g., CXCR3 antagonists) reduces CTL recruitment and BBB disruption in experimental autoimmune encephalomyelitis [151, 152], while the CCL5-CCR5 axis has demonstrated efficacy in hemorrhagic stroke models [153].

### Localized BBB modulation

Focused ultrasound-mediated BBB opening permits site‑specific delivery of immunomodulators, as shown in glioma with enhanced CTL infiltration [154, 155]. Receptor‑targeted nanoparticles (e.g., Angiopep‑2-decorated carriers co‑delivering granzyme B and CpG) further concentrate CTL‑directed agents at the neurovascular interface [156].

### CTLs cytotoxicity Attenuation

Small-molecule inhibitors of perforin and granzyme (e.g., compounds described by Gonzalez Fierro et al., 2023 [124]) selectively dampen CTL-mediated endothelial apoptosis, offering potential adjunctive therapy in Susac’s syndrome and multiple sclerosis.

Integrating these therapeutic avenues within humanized platforms will be essential to achieve durable neuroprotection alongside robust pathogen or tumor clearance.

## Conclusion and further challenges

**Fig. 3:**
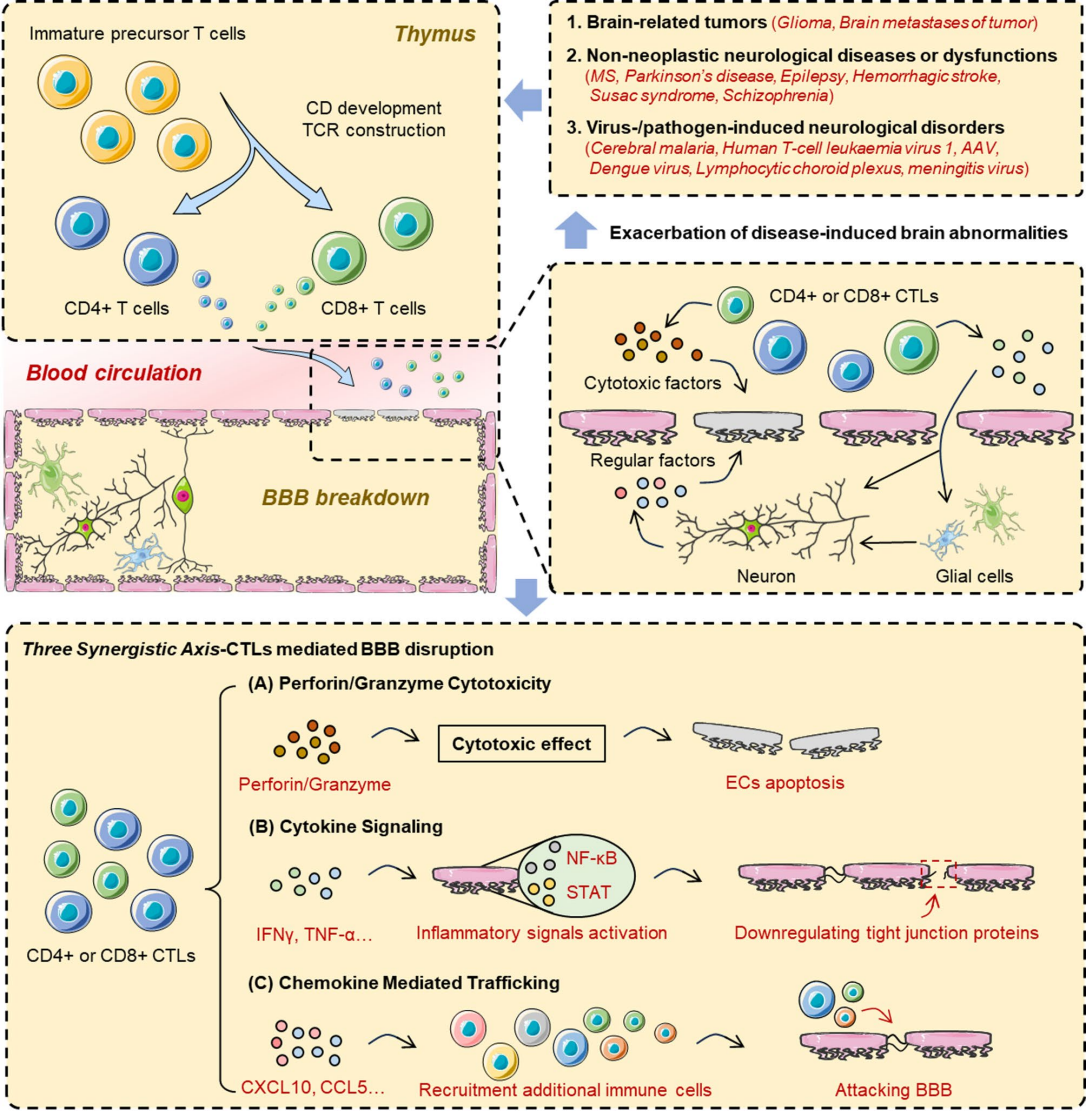
Mechanisms by which CTLs mediate BBB damage. (**A**) Perforin/Granzyme Cytotoxicity: CTLs release perforin and granzyme B, inducing apoptosis of brain microvascular endothelial cells. (**B**) Cytokine Signaling: IFN-γ and TNF-α from CTLs activate JAK/STAT and NF-κB in endothelial cells, downregulating tight junction proteins. (**C**) Chemokine-Mediated Trafficking: CTL-derived CXCL10 and CCL5 establish chemotactic gradients, recruiting CTLs and bystander leukocytes via CXCR3 and CCR5. Abbreviations: CTL, cytotoxic T lymphocyte; BMEC, brain microvascular endothelial cell; IFN-γ, interferon-gamma; TNF-α, tumor necrosis factor-alpha; JAK, Janus kinase; STAT, signal transducer and activator of transcription; NF-κB, nuclear factor kappa-light-chain-enhancer of activated B cells; ICAM-1, intercellular adhesion molecule-1; VCAM-1, vascular cell adhesion molecule-1; MMP, matrix metalloproteinase; CXCL10, C-X-C motif chemokine ligand 10; CCL5, C-C motif chemokine ligand 5; CXCR3, C-X-C motif chemokine receptor 3; CCR5, C-C motif chemokine receptor 5

CTLs exert profound effects on BBB integrity in immune-mediated neurological disorders, including autoimmune diseases and pathogen-induced conditions. Three principal mechanisms have been identified (Fig. 3): (a) Direct cytotoxicity, wherein CTLs deploy perforin and granzyme to induce endothelial apoptosis [157]; (b) Neuron-mediated disruption, via CTL-altered neuronal VEGF production that compromises tight junctions [107]; and (c) Immune-cell facilitation, whereby other leukocytes or resident glia amplify CTL-triggered BBB damage [108, 139]. Additional context-specific pathways, such as HTLV-1 vesicular transmission by CTLs, underscore the complexity of CTL–BBB interactions [134].

To integrate the diverse molecular mechanisms detailed above, we propose a unified model comprising three interlinked axes by which CTLs disrupt BBB integrity: (a) Perforin/Granzyme Cytotoxicity: CTLs release perforin and granzyme B, forming pores in endothelial membranes and activating caspase cascades to induce apoptosis. (b) IFN-γ/TNF-α Signaling: CTL-derived IFN-γ and TNF-α activate JAK/STAT and NF-κB pathways in brain microvascular endothelial cells, downregulating tight junction proteins. (c) Chemokine-Mediated Trafficking: CTLs secrete CXCL10 and CCL5, establishing chemotactic gradients that recruit additional immune cells via CXCR3 and CCR5, promoting diapedesis. These axes converge synergistically to amplify BBB permeability, suggesting that combinatorial therapeutic strategies targeting multiple pathways may enhance barrier preservation.

Despite the beneficial role of activated CTLs, particularly CD8^+^ cells, in targeting pathogens and infected cells in the brain, their potent cytotoxicity often results in collateral damage to healthy cells. Perforin, a major toxic factor, can inadvertently harm normal cells, disrupting the BBB structure, which is primarily composed of brain endothelial cells. Peripheral CTLs must traverse this natural barrier to exert their pathogen-killing function within the brain. Thus, CTL toxicity towards endothelial cells is partly aimed at facilitating brain entry, but this breach can lead to neurological dysfunction. In autoimmune diseases, activated peripheral CTLs also congregate around brain endothelial cells, causing BBB damage and neurological disorders. This is partly due to increased MHC I expression on endothelial cells, which may attract CD8^+^ CTLs [157]. Granzyme B and perforin are primary toxic mediators for CTLs. Research shows that reducing or knocking out perforin expression in mouse disease models protects BBB integrity, improves disease symptoms, and increases survival rates. Therefore, CTLs might be more harmful than beneficial in certain disease stages, and reduced perforin expression could protect the BBB and enhance survival. However, determining when to inhibit or enhance CTLs function requires further investigation.

CD4⁺ CTLs, although less studied, similarly perturb BBB function. We hypothesize that these cells predominantly assist immune responses under homeostatic conditions and may employ non perforin pathways, such as IFN γ/IFNGR1 and SPP1/ITGB1 signaling, to exert cytotoxicity during chronic inflammation. Rigorous validation of these mechanisms is warranted.

The ongoing global COVID-19 pandemic, caused by SARS-CoV-2, persists despite advancements in vaccination and increased natural immunity. Prolonged infection has been linked to brain fog and cognitive impairment, with disruption of the BBB playing a critical role [158, 159]. Research has shown that SARS-CoV-2 infection triggers CD3^+^ T cell infiltration in the hippocampus and brainstem of infected mice [160]. Transcriptomic sequencing of peripheral blood mononuclear cells from COVID-19 patients with cognitive dysfunction also revealed significant enrichment of pathways related to T cell differentiation and activation, as identified through Gene Ontology (GO) analysis [161]. These findings suggest a potential role for T cells, including CTLs, in regulating BBB function during SARS-CoV-2 infection. However, the direct involvement of CTLs and the underlying mechanisms require further investigation.

Collectively, CTLs are pivotal regulators of neurovascular integrity. Future research must integrate high-resolution in vivo imaging, humanized BBB platforms, and single-cell omics to map CTL dynamics and identify targets for selective modulation, thereby preserving barrier function without compromising host defense.

## Electronic Supplementary Material

Below is the link to the electronic supplementary material.

Supplementary Material 1

## References

[1] Sweeney MD, Zhao Z, Montagne A, Nelson AR, Zlokovic BV. Blood-Brain barrier: from physiology to disease and back. Physiol Rev. 2019;99:21–78. 30280653 10.1152/physrev.00050.2017 PMC6335099

[2] Huang J, Ding J, Wang X, Gu C, He Y, Li Y, Fan H, Xie Q, Qi X, Wang Z, Qiu P. Transfer of neuron-derived alpha-synuclein to astrocytes induces neuroinflammation and blood-brain barrier damage after methamphetamine exposure: involving the regulation of nuclear receptor-associated protein 1. Brain Behav Immun. 2022;106:247–61. 36089218 10.1016/j.bbi.2022.09.002

[3] Hou W, Yao J, Liu J, Lin X, Wei J, Yin X, Huang H, Chen X, Yang GY, He X. USP14 Inhibition promotes recovery by protecting BBB integrity and attenuating neuroinflammation in MCAO mice. CNS Neurosci Ther. 2023;29:3612–23. 37269080 10.1111/cns.14292 PMC10580339

[4] Aydin S, Pareja J, Schallenberg VM, Klopstein A, Gruber T, Page N, Bouillet E, Blanchard N, Liblau R, Korbelin J, et al. Antigen recognition detains CD8(+) T cells at the blood-brain barrier and contributes to its breakdown. Nat Commun. 2023;14:3106. 37253744 10.1038/s41467-023-38703-2 PMC10229608

[5] Soldati S, Bar A, Vladymyrov M, Glavin D, McGrath JL, Gosselet F, Nishihara H, Goelz S, Engelhardt B. High levels of endothelial ICAM-1 prohibit natalizumab mediated abrogation of CD4(+) T cell arrest on the inflamed BBB under flow in vitro. J Neuroinflammation. 2023;20:123. 37221552 10.1186/s12974-023-02797-8 PMC10204262

[6] Ahn JJ, Islam Y, Clarkson-Paredes C, Karl MT, Miller RH. B cell depletion modulates glial responses and enhances blood vessel integrity in a model of multiple sclerosis. Neurobiol Dis. 2023;187:106290. 37709209 10.1016/j.nbd.2023.106290

[7] Tang D, Kang R, Coyne CB, Zeh HJ, Lotze MT. PAMPs and damps: signal 0s that spur autophagy and immunity. Immunol Rev. 2012;249:158–75. 22889221 10.1111/j.1600-065X.2012.01146.x PMC3662247

[8] Wik JA, Skalhegg BS. T cell metabolism in infection. Front Immunol. 2022;13:840610. 35359994 10.3389/fimmu.2022.840610 PMC8964062

[9] Eibel H, Kraus H, Sic H, Kienzler AK, Rizzi M. B cell biology: an overview. Curr Allergy Asthma Rep. 2014;14:434. 24633618 10.1007/s11882-014-0434-8

[10] Chapman NM, Boothby MR, Chi H. Metabolic coordination of T cell quiescence and activation. Nat Rev Immunol. 2020;20:55–70. 31406325 10.1038/s41577-019-0203-y

[11] Wu D, Chen Q, Chen X, Han F, Chen Z, Wang Y. The blood-brain barrier: structure, regulation, and drug delivery. Signal Transduct Target Ther. 2023;8:217. 37231000 10.1038/s41392-023-01481-w PMC10212980

[12] Zhao Y, Gan L, Ren L, Lin Y, Ma C, Lin X. Factors influencing the blood-brain barrier permeability. Brain Res. 2022;1788:147937. 35568085 10.1016/j.brainres.2022.147937

[13] Ehrlich P. Das Sauerstoff-Bedürfniss des organismus: eine Farbenanalytische studie. A. Hirschwald. 1885.

[14] Goldmann EE. Die äussere und innere skeretion des Gesunden organismus Im lichte der Vitalen Färbung. H. Laupp’schen Buchhandlung. 1909.

[15] Stern L, Gautier R. Recherches Sur Le Liquide céphalo-rachidien: I.–Les rapports entre Le Liquide céphalo-rachidien et La circulation sanguine. Archives Internationales De Physiologie. 1921;17:138–92.

[16] Stewart PA, Wiley MJ. Developing nervous tissue induces formation of blood-brain barrier characteristics in invading endothelial cells: a study using quail–chick transplantation chimeras. Dev Biol. 1981;84:183–92. 7250491 10.1016/0012-1606(81)90382-1

[17] Harati R, Villegier AS, Banks WA, Mabondzo A. Susceptibility of juvenile and adult blood-brain barrier to endothelin-1: regulation of P-glycoprotein and breast cancer resistance protein expression and transport activity. J Neuroinflammation. 2012;9:273. 23253775 10.1186/1742-2094-9-273 PMC3547749

[18] Cai Z, Qiao PF, Wan CQ, Cai M, Zhou NK, Li Q. Role of Blood-Brain barrier in alzheimer’s disease. J Alzheimers Dis. 2018;63:1223–34. 29782323 10.3233/JAD-180098

[19] Eichler AF, Chung E, Kodack DP, Loeffler JS, Fukumura D, Jain RK. The biology of brain metastases-translation to new therapies. Nat Rev Clin Oncol. 2011;8:344–56. 21487419 10.1038/nrclinonc.2011.58 PMC3259742

[20] Saraiva C, Praca C, Ferreira R, Santos T, Ferreira L, Bernardino L. Nanoparticle-mediated brain drug delivery: overcoming blood-brain barrier to treat neurodegenerative diseases. J Control Release. 2016;235:34–47. 27208862 10.1016/j.jconrel.2016.05.044

[21] Segarra M, Aburto MR, Acker-Palmer A. Blood-Brain barrier dynamics to maintain brain homeostasis. Trends Neurosci. 2021;44:393–405. 33423792 10.1016/j.tins.2020.12.002

[22] Fong CW. Permeability of the Blood-Brain barrier: molecular mechanism of transport of drugs and physiologically important compounds. J Membr Biol. 2015;248:651–69. 25675910 10.1007/s00232-015-9778-9

[23] Galea I. The blood-brain barrier in systemic infection and inflammation. Cell Mol Immunol. 2021;18:2489–501. 34594000 10.1038/s41423-021-00757-x PMC8481764

[24] Murphy K, Weaver C. Janeway’s immunobiology. Garland science. 2016.

[25] Schmidt ME, Varga SM. The CD8 T cell response to respiratory virus infections. Front Immunol. 2018;9:678. 29686673 10.3389/fimmu.2018.00678 PMC5900024

[26] Takeuchi A, Saito T. CD4 CTL, a cytotoxic subset of CD4(+) T Cells, their differentiation and function. Front Immunol. 2017;8:194. 28280496 10.3389/fimmu.2017.00194 PMC5321676

[27] Martens A, Wistuba-Hamprecht K, Yuan J, Postow MA, Wong P, Capone M, Madonna G, Khammari A, Schilling B, Sucker A, et al. Increases in absolute lymphocytes and Circulating CD4 + and CD8 + T cells are associated with positive clinical outcome of melanoma patients treated with ipilimumab. Clin Cancer Res. 2016;22:4848–58. 27169993 10.1158/1078-0432.CCR-16-0249 PMC5544386

[28] Quezada SA, Simpson TR, Peggs KS, Merghoub T, Vider J, Fan X, Blasberg R, Yagita H, Muranski P, Antony PA, et al. Tumor-reactive CD4(+) T cells develop cytotoxic activity and eradicate large established melanoma after transfer into lymphopenic hosts. J Exp Med. 2010;207:637–50. 20156971 10.1084/jem.20091918 PMC2839156

[29] Zhou X, Tang J, Cao H, Fan H, Li B. Tissue resident regulatory T cells: novel therapeutic targets for human disease. Cell Mol Immunol. 2015;12:543–52. 25891216 10.1038/cmi.2015.23 PMC4579654

[30] Georgiev P, Charbonnier LM, Chatila TA. Regulatory T cells: the many faces of Foxp3. J Clin Immunol. 2019;39:623–40. 31478130 10.1007/s10875-019-00684-7 PMC6754763

[31] Sakaguchi S, Yamaguchi T, Nomura T, Ono M. Regulatory T cells and immune tolerance. Cell. 2008;133:775–87. 18510923 10.1016/j.cell.2008.05.009

[32] Ho P, Cahir-McFarland E, Fontenot JD, Lodie T, Nada A, Tang Q, Turka LA, Bluestone JA. Harnessing regulatory T cells to Establish immune tolerance. Sci Transl Med. 2024;16:eadm8859. 38478632 10.1126/scitranslmed.adm8859

[33] Togashi Y, Shitara K, Nishikawa H. Regulatory T cells in cancer immunosuppression - implications for anticancer therapy. Nat Rev Clin Oncol. 2019;16:356–71. 30705439 10.1038/s41571-019-0175-7

[34] Zhu J, Yamane H, Paul WE. Differentiation of effector CD4 T cell populations (*). Annu Rev Immunol. 2010;28:445–89. 20192806 10.1146/annurev-immunol-030409-101212 PMC3502616

[35] Komai T, Inoue M, Okamura T, Morita K, Iwasaki Y, Sumitomo S, Shoda H, Yamamoto K, Fujio K. Transforming growth Factor-beta and Interleukin-10 synergistically regulate humoral immunity via modulating metabolic signals. Front Immunol. 2018;9:1364. 29963056 10.3389/fimmu.2018.01364 PMC6010538

[36] McGinley AM, Edwards SC, Raverdeau M, Mills KHG. Th17 cells, gammadelta T cells and their interplay in EAE and multiple sclerosis. J Autoimmun. 2018. 10.1016/j.jaut.2018.01.001 29395738

[37] Derkow K, Kruger C, Dembny P, Lehnardt S. Microglia induce neurotoxic IL-17 + gammadelta T cells dependent on TLR2, TLR4, and TLR9 activation. PLoS ONE. 2015;10:e0135898. 26288016 10.1371/journal.pone.0135898 PMC4545749

[38] Verreycken J, Baeten P, Broux B. Regulatory T cell therapy for multiple sclerosis: breaching (blood-brain) barriers. Hum Vaccin Immunother. 2022;18:2153534. 36576251 10.1080/21645515.2022.2153534 PMC9891682

[39] Caplan HW, Prabhakara KS, Toledano Furman NE, Zorofchian S, Martin C, Xue H, Olson SD, Cox CS Jr. Human-derived Treg and MSC combination therapy May augment immunosuppressive potency in vitro, but did not improve blood brain barrier integrity in an experimental rat traumatic brain injury model. PLoS ONE. 2021;16:e0251601. 34038436 10.1371/journal.pone.0251601 PMC8153465

[40] Brazin KN, Mallis RJ, Das DK, Feng Y, Hwang W, Wang JH, Wagner G, Lang MJ, Reinherz EL. Structural features of the alphabetatcr mechanotransduction apparatus that promote pMHC discrimination. Front Immunol. 2015;6:441. 26388869 10.3389/fimmu.2015.00441 PMC4558533

[41] Dustin ML. The immunological synapse. Cancer Immunol Res. 2014;2:1023–33. 25367977 10.1158/2326-6066.CIR-14-0161 PMC4692051

[42] Bettini ML, Guy C, Dash P, Vignali KM, Hamm DE, Dobbins J, Gagnon E, Thomas PG, Wucherpfennig KW, Vignali DA. Membrane association of the CD3epsilon signaling domain is required for optimal T cell development and function. J Immunol. 2014;193:258–67. 24899501 10.4049/jimmunol.1400322 PMC4065803

[43] Zumerle S, Molon B, Viola A. Membrane rafts in T cell activation: A spotlight on CD28 costimulation. Front Immunol. 2017;8:1467. 29163534 10.3389/fimmu.2017.01467 PMC5675840

[44] Faia K, White K, Murphy E, Proctor J, Pink M, Kosmider N, McGovern K, Kutok J. The phosphoinositide-3 kinase (PI3K)-delta,gamma inhibitor, Duvelisib shows preclinical synergy with multiple targeted therapies in hematologic malignancies. PLoS ONE. 2018;13:e0200725. 30067771 10.1371/journal.pone.0200725 PMC6070190

[45] Raskov H, Orhan A, Christensen JP, Gogenur I. Cytotoxic CD8(+) T cells in cancer and cancer immunotherapy. Br J Cancer. 2021;124:359–67. 32929195 10.1038/s41416-020-01048-4 PMC7853123

[46] Fu Q, Fu TM, Cruz AC, Sengupta P, Thomas SK, Wang S, Siegel RM, Wu H, Chou JJ. Structural basis and functional role of intramembrane trimerization of the Fas/CD95 death receptor. Mol Cell. 2016;61:602–13. 26853147 10.1016/j.molcel.2016.01.009 PMC4761300

[47] Basu R, Whitlock BM, Husson J, Le Floc’h A, Jin W, Oyler-Yaniv A, Dotiwala F, Giannone G, Hivroz C, Biais N, et al. Cytotoxic T cells use mechanical force to potentiate target cell killing. Cell. 2016;165:100–10. 26924577 10.1016/j.cell.2016.01.021 PMC4808403

[48] Gordy C, He YW. Endocytosis by target cells: an essential means for perforin- and granzyme-mediated killing. Cell Mol Immunol. 2012;9:5–6. 21927017 10.1038/cmi.2011.45 PMC4002933

[49] Bryceson YT, Chiang SC, Darmanin S, Fauriat C, Schlums H, Theorell J, Wood SM. Molecular mechanisms of natural killer cell activation. J Innate Immun. 2011;3:216–26. 21454962 10.1159/000325265

[50] Tamzalit F, Tran D, Jin W, Boyko V, Bazzi H, Kepecs A, Kam LC, Anderson KV, Huse M. Centrioles control the capacity, but not the specificity, of cytotoxic T cell killing. Proc Natl Acad Sci U S A. 2020;117:4310–9. 32041868 10.1073/pnas.1913220117 PMC7049148

[51] Luik RM, Wu MM, Buchanan J, Lewis RS. The elementary unit of store-operated Ca2 + entry: local activation of CRAC channels by STIM1 at ER-plasma membrane junctions. J Cell Biol. 2006;174:815–25. 16966423 10.1083/jcb.200604015 PMC2064336

[52] Lioudyno MI, Kozak JA, Penna A, Safrina O, Zhang SL, Sen D, Roos J, Stauderman KA, Cahalan MD. Orai1 and STIM1 move to the immunological synapse and are up-regulated during T cell activation. Proc Natl Acad Sci U S A. 2008;105:2011–6. 18250319 10.1073/pnas.0706122105 PMC2538873

[53] Vaeth M, Kahlfuss S, Feske S. CRAC channels and calcium signaling in T Cell-Mediated immunity. Trends Immunol. 2020;41:878–901. 32711944 10.1016/j.it.2020.06.012 PMC7985820

[54] Quintana A, Kummerow C, Junker C, Becherer U, Hoth M. Morphological changes of T cells following formation of the immunological synapse modulate intracellular calcium signals. Cell Calcium. 2009;45:109–22. 18789821 10.1016/j.ceca.2008.07.003

[55] Schwindling C, Quintana A, Krause E, Hoth M. Mitochondria positioning controls local calcium influx in T cells. J Immunol. 2010;184:184–90. 19949095 10.4049/jimmunol.0902872

[56] Quintana A, Pasche M, Junker C, Al-Ansary D, Rieger H, Kummerow C, Nunez L, Villalobos C, Meraner P, Becherer U, et al. Calcium microdomains at the immunological synapse: how ORAI channels, mitochondria and calcium pumps generate local calcium signals for efficient T-cell activation. EMBO J. 2011;30:3895–912. 21847095 10.1038/emboj.2011.289 PMC3209779

[57] Babich A, Burkhardt JK. Coordinate control of cytoskeletal remodeling and calcium mobilization during T-cell activation. Immunol Rev. 2013;256:80–94. 24117814 10.1111/imr.12123 PMC3824381

[58] Halimani M, Pattu V, Marshall MR, Chang HF, Matti U, Jung M, Becherer U, Krause E, Hoth M, Schwarz EC, Rettig J. Syntaxin11 serves as a t-SNARE for the fusion of lytic granules in human cytotoxic T lymphocytes. Eur J Immunol. 2014;44:573–84. 24227526 10.1002/eji.201344011

[59] Marshall MR, Pattu V, Halimani M, Maier-Peuschel M, Muller ML, Becherer U, Hong W, Hoth M, Tschernig T, Bryceson YT, Rettig J. VAMP8-dependent fusion of recycling endosomes with the plasma membrane facilitates T lymphocyte cytotoxicity. J Cell Biol. 2015;210:135–51. 26124288 10.1083/jcb.201411093 PMC4493996

[60] Chang HF, Schirra C, Pattu V, Krause E, Becherer U. Lytic granule exocytosis at immune synapses: lessons from neuronal synapses. Front Immunol. 2023;14:1177670. 37275872 10.3389/fimmu.2023.1177670 PMC10233144

[61] Dornmair K, Goebels N, Weltzien HU, Wekerle H, Hohlfeld R. T-cell-mediated autoimmunity: novel techniques to characterize autoreactive T-cell receptors. Am J Pathol. 2003;163:1215–26. 14507631 10.1016/S0002-9440(10)63481-5 PMC1868314

[62] Shevchenko I, Bazhin AV. Metabolic checkpoints: novel avenues for immunotherapy of cancer. Front Immunol. 2018;9:1816. 30131808 10.3389/fimmu.2018.01816 PMC6090142

[63] Joller N, Kuchroo VK. Tim-3, Lag-3, and TIGIT. Curr Top Microbiol Immunol. 2017;410:127–56. 28900677 10.1007/82_2017_62 PMC5902028

[64] Amatore F, Gorvel L, Olive D. Inducible Co-Stimulator (ICOS) as a potential therapeutic target for anti-cancer therapy. Expert Opin Ther Targets. 2018;22:343–51. 29468927 10.1080/14728222.2018.1444753

[65] Bethmann D, Feng Z, Fox BA. Immunoprofiling as a predictor of patient’s response to cancer therapy-promises and challenges. Curr Opin Immunol. 2017;45:60–72. 28222333 10.1016/j.coi.2017.01.005

[66] Havel JJ, Chowell D, Chan TA. The evolving landscape of biomarkers for checkpoint inhibitor immunotherapy. Nat Rev Cancer. 2019;19:133–50. 30755690 10.1038/s41568-019-0116-x PMC6705396

[67] Schmidt EV. Developing combination strategies using PD-1 checkpoint inhibitors to treat cancer. Semin Immunopathol. 2019;41:21–30. 30374524 10.1007/s00281-018-0714-9 PMC6323091

[68] Souza-Fonseca-Guimaraes F, Cursons J, Huntington ND. The emergence of natural killer cells as a major target in cancer immunotherapy. Trends Immunol. 2019;40:142–58. 30639050 10.1016/j.it.2018.12.003

[69] Motzer RJ, Penkov K, Haanen J, Rini B, Albiges L, Campbell MT, Venugopal B, Kollmannsberger C, Negrier S, Uemura M, et al. Avelumab plus axitinib versus Sunitinib for advanced Renal-Cell carcinoma. N Engl J Med. 2019;380:1103–15. 30779531 10.1056/NEJMoa1816047 PMC6716603

[70] Overman MJ, Lonardi S, Wong KYM, Lenz HJ, Gelsomino F, Aglietta M, Morse MA, Van Cutsem E, McDermott R, Hill A, et al. Durable clinical benefit with nivolumab plus ipilimumab in DNA mismatch Repair-Deficient/Microsatellite Instability-High metastatic colorectal cancer. J Clin Oncol. 2018;36:773–9. 29355075 10.1200/JCO.2017.76.9901

[71] Brien JD, Uhrlaub JL, Nikolich-Zugich J. West nile virus-specific CD4 T cells exhibit direct antiviral cytokine secretion and cytotoxicity and are sufficient for antiviral protection. J Immunol. 2008;181:8568–75. 19050276 10.4049/jimmunol.181.12.8568 PMC3504655

[72] Yeh JH, Sidhu SS, Chan AC. Regulation of a late phase of T cell Polarity and effector functions by Crtam. Cell. 2008;132:846–59. 18329370 10.1016/j.cell.2008.01.013

[73] Jones DM, Tuazon JA, Read KA, Leonard MR, Pokhrel S, Sreekumar BK, Warren RT, Yount JS, Collins PL, Oestreich KJ. Cytotoxic programming of CD4 + T cells is regulated by opposing actions of the related transcription factors Eos and Aiolos. J Immunol. 2024;212:1129–41. 38363226 10.4049/jimmunol.2300748 PMC10948294

[74] Donnarumma T, Young GR, Merkenschlager J, Eksmond U, Bongard N, Nutt SL, Boyer C, Dittmer U, Le-Trilling VT, Trilling M, et al. Opposing development of cytotoxic and follicular helper CD4 T cells controlled by the TCF-1-Bcl6 nexus. Cell Rep. 2016;17:1571–83. 27806296 10.1016/j.celrep.2016.10.013 PMC5149578

[75] Roselli E, Boucher JC, Li G, Kotani H, Spitler K, Reid K, Cervantes EV, Bulliard Y, Tu N, Lee SB, et al. 4-1BB and optimized CD28 co-stimulation enhances function of human mono-specific and bi-specific third-generation CAR T cells. J Immunother Cancer. 2021;9. 10.1136/jitc-2021-003354 PMC8552146 34706886

[76] Barber DL, Mayer-Barber KD, Feng CG, Sharpe AH, Sher A. CD4 T cells promote rather than control tuberculosis in the absence of PD-1-mediated Inhibition. J Immunol. 2011;186:1598–607. 21172867 10.4049/jimmunol.1003304 PMC4059388

[77] Jayaraman P, Jacques MK, Zhu C, Steblenko KM, Stowell BL, Madi A, Anderson AC, Kuchroo VK, Behar SM. TIM3 mediates T cell exhaustion during Mycobacterium tuberculosis infection. PLoS Pathog. 2016;12:e1005490. 26967901 10.1371/journal.ppat.1005490 PMC4788425

[78] Malyshkina A, Bruggemann A, Paschen A, Dittmer U. Cytotoxic CD4(+) T cells in chronic viral infections and cancer. Front Immunol. 2023;14:1271236. 37965314 10.3389/fimmu.2023.1271236 PMC10642198

[79] Carpenter AC, Bosselut R. Decision checkpoints in the thymus. Nat Immunol. 2010;11:666–73. 20644572 10.1038/ni.1887 PMC3388799

[80] Billings P, Burakoff S, Dorf ME, Benacerraf B. Cytotoxic T lymphocytes specific for I region determinants do not require interactions with H-2K or D gene products. J Exp Med. 1977;145:1387–92. 67179 10.1084/jem.145.5.1387 PMC2180671

[81] Krensky AM, Reiss CS, Mier JW, Strominger JL, Burakoff SJ. Long-term human cytolytic T-cell lines allospecific for HLA-DR6 antigen are OKT4+. Proc Natl Acad Sci U S A. 1982;79:2365–9. 6980419 10.1073/pnas.79.7.2365 PMC346194

[82] Reis BS, Rogoz A, Costa-Pinto FA, Taniuchi I, Mucida D. Mutual expression of the transcription factors Runx3 and ThPOK regulates intestinal CD4(+) T cell immunity. Nat Immunol. 2013;14:271–80. 23334789 10.1038/ni.2518 PMC3804366

[83] Takeuchi A, Badr Mel S, Miyauchi K, Ishihara C, Onishi R, Guo Z, Sasaki Y, Ike H, Takumi A, Tsuji NM, et al. CRTAM determines the CD4 + cytotoxic T lymphocyte lineage. J Exp Med. 2016;213:123–38. 26694968 10.1084/jem.20150519 PMC4710199

[84] Trapani JA, Smyth MJ. Functional significance of the perforin/granzyme cell death pathway. Nat Rev Immunol. 2002;2:735–47. 12360212 10.1038/nri911

[85] Hildemann SK, Eberlein J, Davenport B, Nguyen TT, Victorino F, Homann D. High efficiency of antiviral CD4(+) killer T cells. PLoS ONE. 2013;8:e60420. 23565245 10.1371/journal.pone.0060420 PMC3614903

[86] Appay V, Zaunders JJ, Papagno L, Sutton J, Jaramillo A, Waters A, Easterbrook P, Grey P, Smith D, McMichael AJ, et al. Characterization of CD4(+) CTLs ex vivo. J Immunol. 2002;168:5954–8. 12023402 10.4049/jimmunol.168.11.5954

[87] Preglej T, Hamminger P, Luu M, Bulat T, Andersen L, Goschl L, Stolz V, Rica R, Sandner L, Waltenberger D, et al. Histone deacetylases 1 and 2 restrain CD4 + cytotoxic T lymphocyte differentiation. JCI Insight. 2020;5. 10.1172/jci.insight.133393 PMC7101144 32102981

[88] Patil VS, Madrigal A, Schmiedel BJ, Clarke J, O’Rourke P, de Silva AD, Harris E, Peters B, Seumois G, Weiskopf D, et al. Precursors of human CD4(+) cytotoxic T lymphocytes identified by single-cell transcriptome analysis. Sci Immunol. 2018;3. 10.1126/sciimmunol.aan8664 PMC5931334 29352091

[89] Knudson CJ, Ferez M, Alves-Peixoto P, Erkes DA, Melo-Silva CR, Tang L, Snyder CM, Sigal LJ. Mechanisms of antiviral cytotoxic CD4 T cell differentiation. J Virol. 2021;95:e0056621. 34260270 10.1128/JVI.00566-21 PMC8428409

[90] Fang M, Siciliano NA, Hersperger AR, Roscoe F, Hu A, Ma X, Shamsedeen AR, Eisenlohr LC, Sigal LJ. Perforin-dependent CD4 + T-cell cytotoxicity contributes to control a murine poxvirus infection. Proc Natl Acad Sci U S A. 2012;109:9983–8. 22665800 10.1073/pnas.1202143109 PMC3382508

[91] Miggelbrink AM, Jackson JD, Lorrey SJ, Srinivasan ES, Waibl-Polania J, Wilkinson DS, Fecci PE. CD4 T-Cell exhaustion: does it exist and what are its roles in cancer? Clin Cancer Res. 2021;27:5742–52. 34127507 10.1158/1078-0432.CCR-21-0206 PMC8563372

[92] Flores-Gonzalez J, Ramon-Luing LA, Falfan-Valencia R, Batista CVF, Soto-Alvarez S, Huerta-Nunez L, Chavez-Galan L. The presence of cytotoxic CD4 and exhausted-like CD8 + T-cells is a signature of active tuberculosis. Biochim Biophys Acta Mol Basis Dis. 2024;1870:167219. 38734321 10.1016/j.bbadis.2024.167219

[93] Wyde PR, Gilbert BE, Levy BM. Evidence that T-lymphocytes are part of the blood-brain barrier to virus dissemination. J Neuroimmunol. 1983;5:47–58. 6603472 10.1016/0165-5728(83)90025-5

[94] Hafler DA, Weiner HL. T cells in multiple sclerosis and inflammatory central nervous system diseases. Immunol Rev. 1987;100:307–32. 3326824 10.1111/j.1600-065x.1987.tb00537.x

[95] Hafler DA, Weiner HL. In vivo labeling of blood T cells: rapid traffic into cerebrospinal fluid in multiple sclerosis. Ann Neurol. 1987;22:89–93. 3498435 10.1002/ana.410220121

[96] Gordon LB, Nolan SC, Cserr HF, Knopf PM, Harling-Berg CJ. Growth of P511 mastocytoma cells in BALB/c mouse brain elicits CTL response without tumor elimination: a new tumor model for regional central nervous system immunity. J Immunol. 1997;159:2399–408. 9278331

[97] Chen PY, Hsieh HY, Huang CY, Lin CY, Wei KC, Liu HL. Focused ultrasound-induced blood-brain barrier opening to enhance interleukin-12 delivery for brain tumor immunotherapy: a preclinical feasibility study. J Transl Med. 2015;13:93. 25784614 10.1186/s12967-015-0451-y PMC4369363

[98] Angelova AL, Barf M, Geletneky K, Unterberg A, Rommelaere J. Immunotherapeutic potential of oncolytic H-1 parvovirus: hints of glioblastoma microenvironment conversion towards immunogenicity. Viruses. 2017;9. 10.3390/v9120382 PMC5744156 29244745

[99] Geletneky K, Hajda J, Angelova AL, Leuchs B, Capper D, Bartsch AJ, Neumann JO, Schoning T, Husing J, Beelte B, et al. Oncolytic H-1 parvovirus shows safety and signs of Immunogenic activity in a first phase I/IIa glioblastoma trial. Mol Ther. 2017;25:2620–34. 28967558 10.1016/j.ymthe.2017.08.016 PMC5768665

[100] Peng Y, Zhan M, Karpus A, Zou Y, Mignani S, Majoral JP, Shi X, Shen M. Brain delivery of biomimetic phosphorus Dendrimer/Antibody nanocomplexes for enhanced glioma immunotherapy via immune modulation of T cells and natural killer cells. ACS Nano. 2024;18:10142–55. 38526307 10.1021/acsnano.3c13088

[101] Wang W, Zhang M, Zhang Q, Mohammadniaei M, Shen J, Sun Y. Brain-targeted antigen-generating nanoparticles improve glioblastoma prognosis. J Control Release. 2022;352:399–410. 36309097 10.1016/j.jconrel.2022.10.037

[102] Wei J, Wu D, Shao Y, Guo B, Jiang J, Chen J, Zhang J, Meng F, Zhong Z. ApoE-mediated systemic nanodelivery of granzyme B and CpG for enhanced glioma immunotherapy. J Control Release. 2022;347:68–77. 35513207 10.1016/j.jconrel.2022.04.048

[103] Pohl-Guimaraes F, Yang C, Dyson KA, Wildes TJ, Drake J, Huang J, Flores C, Sayour EJ, Mitchell DA. RNA-Modified T cells mediate effective delivery of Immunomodulatory cytokines to brain tumors. Mol Ther. 2019;27:837–49. 30448196 10.1016/j.ymthe.2018.10.007 PMC6453546

[104] Wang X, Xiong Z, Liu Z, Huang X, Jiang X. Angiopep-2/IP10-EGFRvIIIscFv modified nanoparticles and CTL synergistically inhibit malignant glioblastoma. Sci Rep. 2018;8:12827. 30150691 10.1038/s41598-018-30072-x PMC6110710

[105] Tsukada N, Matsuda M, Miyagi K, Yanagisawa N. Cytotoxicity of T cells for cerebral endothelium in multiple sclerosis. J Neurol Sci. 1993;117:140–7. 8410048 10.1016/0022-510x(93)90166-v

[106] Suidan GL, McDole JR, Chen Y, Pirko I, Johnson AJ. Induction of blood brain barrier tight junction protein alterations by CD8 T cells. PLoS ONE. 2008;3:e3037. 18725947 10.1371/journal.pone.0003037 PMC2516328

[107] Suidan GL, Dickerson JW, Chen Y, McDole JR, Tripathi P, Pirko I, Seroogy KB, Johnson AJ. CD8 T cell-initiated vascular endothelial growth factor expression promotes central nervous system vascular permeability under neuroinflammatory conditions. J Immunol. 2010;184:1031–40. 20008293 10.4049/jimmunol.0902773 PMC2896014

[108] Suidan GL, Dickerson JW, Johnson HL, Chan TW, Pavelko KD, Pirko I, Seroogy KB, Johnson AJ. Preserved vascular integrity and enhanced survival following neuropilin-1 Inhibition in a mouse model of CD8 T cell-initiated CNS vascular permeability. J Neuroinflammation. 2012;9:218. 22985494 10.1186/1742-2094-9-218 PMC3489603

[109] Schneider R, Mohebiany AN, Ifergan I, Beauseigle D, Duquette P, Prat A, Arbour N. B cell-derived IL-15 enhances CD8 T cell cytotoxicity and is increased in multiple sclerosis patients. J Immunol. 2011;187:4119–28. 21911607 10.4049/jimmunol.1100885 PMC5052068

[110] Elkhodiry AA, Zamzam DA, El Tayebi HM. miR-155 and functional proteins of CD8 + T cells as potential prognostic biomarkers for relapsing-remitting multiple sclerosis. Mult Scler Relat Disord. 2021;53:103078. 34171684 10.1016/j.msard.2021.103078

[111] Kooij G, Kroon J, Paul D, Reijerkerk A, Geerts D, van der Pol SM, van Het Hof B, Drexhage JA, van Vliet SJ, Hekking LH, et al. P-glycoprotein regulates trafficking of CD8(+) T cells to the brain parenchyma. Acta Neuropathol. 2014;127:699–711. 24429546 10.1007/s00401-014-1244-8

[112] Raulet DH. Roles of the NKG2D immunoreceptor and its ligands. Nat Rev Immunol. 2003;3:781–90. 14523385 10.1038/nri1199

[113] Ruck T, Bittner S, Gross CC, Breuer J, Albrecht S, Korr S, Gobel K, Pankratz S, Henschel CM, Schwab N, et al. CD4 + NKG2D + T cells exhibit enhanced migratory and encephalitogenic properties in neuroinflammation. PLoS ONE. 2013;8:e81455. 24282598 10.1371/journal.pone.0081455 PMC3839937

[114] Poewe W, Seppi K, Tanner CM, Halliday GM, Brundin P, Volkmann J, Schrag AE, Lang AE. Parkinson disease. Nat Rev Dis Primers. 2017;3:17013. 28332488 10.1038/nrdp.2017.13

[115] Yan S, Si Y, Zhou W, Cheng R, Wang P, Wang D, Ding W, Shi W, Jiang Q, Yang F, Yao L. Single-cell transcriptomics reveals the interaction between peripheral CD4(+) CTLs and mesencephalic endothelial cells mediated by IFNG in parkinson’s disease. Comput Biol Med. 2023;158:106801. 36989741 10.1016/j.compbiomed.2023.106801

[116] Marchi N, Johnson AJ, Puvenna V, Johnson HL, Tierney W, Ghosh C, Cucullo L, Fabene PF, Janigro D. Modulation of peripheral cytotoxic cells and ictogenesis in a model of seizures. Epilepsia. 2011;52:1627–34. 21627645 10.1111/j.1528-1167.2011.03080.x PMC3728674

[117] Johnson HL, Jin F, Pirko I, Johnson AJ. Theiler’s murine encephalomyelitis virus as an experimental model system to study the mechanism of blood-brain barrier disruption. J Neurovirol. 2014;20:107–12. 23857332 10.1007/s13365-013-0187-5 PMC3894260

[118] Xie P, Zhu S, Zhou H, Fang R, Zhuang J, Wen J, Yang M, He J. Rapamycin plays an Anti-Epileptic role by restoring Blood-Brain barrier Dysfunction, balancing T cell subsets and inhibiting neuronal apoptosis. Discov Med. 2023;35:1043–51. 38058069 10.24976/Discov.Med.202335179.100

[119] Zhou S, Liu C, Wang J, Ye J, Lian Q, Gan L, Deng S, Xu T, Guo Y, Li W, et al. CCL5 mediated astrocyte-T cell interaction disrupts blood-brain barrier in mice after hemorrhagic stroke. J Cereb Blood Flow Metab. 2024;44:367–83. 37974301 10.1177/0271678X231214838 PMC10870968

[120] Hardy TA, O’Brien B, Gerbis N, Barnett MH, Reddel SW, Brewer J, Herkes GK, Silberstein P, Garsia RJ, Watson JD, et al. Brain histopathology in three cases of susac’s syndrome: implications for lesion pathogenesis and treatment. J Neurol Neurosurg Psychiatry. 2015;86:582–4. 25168394 10.1136/jnnp-2014-308240

[121] Magro CM, Poe JC, Lubow M, Susac JO. Susac syndrome: an organ-specific autoimmune endotheliopathy syndrome associated with anti-endothelial cell antibodies. Am J Clin Pathol. 2011;136:903–12. 22095376 10.1309/AJCPERI7LC4VNFYK

[122] Susac JO, Egan RA, Rennebohm RM, Lubow M. Susac’s syndrome: 1975–2005 microangiopathy/autoimmune endotheliopathy. J Neurol Sci. 2007;257:270–2. 17331544 10.1016/j.jns.2007.01.036

[123] Gross CC, Meyer C, Bhatia U, Yshii L, Kleffner I, Bauer J, Troscher AR, Schulte-Mecklenbeck A, Herich S, Schneider-Hohendorf T, et al. CD8(+) T cell-mediated endotheliopathy is a targetable mechanism of neuro-inflammation in Susac syndrome. Nat Commun. 2019;10:5779. 31852955 10.1038/s41467-019-13593-5 PMC6920411

[124] Gonzalez-Fierro C, Fonte C, Dufourd E, Cazaentre V, Aydin S, Engelhardt B, Caspi RR, Xu B, Martin-Blondel G, Spicer JA, et al. Effects of a Small-Molecule Perforin inhibitor in a mouse model of CD8 T Cell-Mediated neuroinflammation. Neurol Neuroimmunol Neuroinflamm. 2023;10. 10.1212/NXI.0000000000200117 PMC10119812 37080596

[125] Muller N, Riedel M, Hadjamu M, Schwarz MJ, Ackenheil M, Gruber R. Increase in expression of adhesion molecule receptors on T helper cells during antipsychotic treatment and relationship to blood-brain barrier permeability in schizophrenia. Am J Psychiatry. 1999;156:634–6. 10200747

[126] Potter S, Chan-Ling T, Ball HJ, Mansour H, Mitchell A, Maluish L, Hunt NH. Perforin mediated apoptosis of cerebral microvascular endothelial cells during experimental cerebral malaria. Int J Parasitol. 2006;36:485–96. 16500656 10.1016/j.ijpara.2005.12.005

[127] Huggins MA, Johnson HL, Jin F, A NS, Hanson LM, LaFrance SJ, Butler NS, Harty JT, Johnson AJ. Perforin expression by CD8 T cells is sufficient to cause fatal brain edema during experimental cerebral malaria. Infect Immun. 2017;85. 10.1128/IAI.00985-16 PMC5400849 28264905

[128] Howland SW, Poh CM, Gun SY, Claser C, Malleret B, Shastri N, Ginhoux F, Grotenbreg GM, Renia L. Brain microvessel cross-presentation is a hallmark of experimental cerebral malaria. EMBO Mol Med. 2013;5:984–99. 23681698 10.1002/emmm.201202273 PMC3721469

[129] Wei X, Li Y, Sun X, Zhu X, Feng Y, Liu J, Jiang Y, Shang H, Cui L, Cao Y. Erythropoietin protects against murine cerebral malaria through actions on host cellular immunity. Infect Immun. 2014;82:165–73. 24126529 10.1128/IAI.00929-13 PMC3911832

[130] Kuehlwein JM, Borsche M, Korir PJ, Risch F, Mueller AK, Hubner MP, Hildner K, Hoerauf A, Dunay IR, Schumak B. Protection of Batf3-deficient mice from experimental cerebral malaria correlates with impaired cytotoxic T-cell responses and immune regulation. Immunology. 2020;159:193–204. 31631339 10.1111/imm.13137 PMC6954726

[131] Scheunemann JF, Reichwald JJ, Korir PJ, Kuehlwein JM, Jenster LM, Hammerschmidt-Kamper C, Lewis MD, Klocke K, Borsche M, Schwendt KE, et al. Eosinophils suppress the migration of T cells into the brain of plasmodium berghei-Infected Ifnar1(-/-) mice and protect them from experimental cerebral malaria. Front Immunol. 2021;12:711876. 34659202 10.3389/fimmu.2021.711876 PMC8514736

[132] Sharma I, Kataria P, Das J. Cerebral malaria pathogenesis: dissecting the role of CD4(+) and CD8(+) T-cells as major effectors in disease pathology. Int Rev Immunol. 2024:1–18. 10.1080/08830185.2024.2336539 38618863

[133] Yamano Y, Nagai M, Brennan M, Mora CA, Soldan SS, Tomaru U, Takenouchi N, Izumo S, Osame M, Jacobson S. Correlation of human T-cell lymphotropic virus type 1 (HTLV-1) mRNA with proviral DNA load, virus-specific CD8(+) T cells, and disease severity in HTLV-1-associated myelopathy (HAM/TSP). Blood. 2002;99:88–94. 11756157 10.1182/blood.v99.1.88

[134] Mor-Vaknin N, Turgeman H, Torgeman A, Wolfson M, Huleihel M, Aboud M. Rapid syncytium formation between human T-cell leukaemia virus type-I (HTLV-I)-infected T-cells and human nervous system cells: a possible implication for tropical spastic paraparesis/HTLV-I associated myelopathy. Cell Biol Int. 1998;22:95–103. 9878096 10.1006/cbir.1998.0241

[135] Ma G, Yasunaga JI, Ohshima K, Matsumoto T, Matsuoka M. Pentosan polysulfate demonstrates Anti-human T-Cell leukemia virus type 1 activities in vitro and in vivo. J Virol. 2019;93. 10.1128/JVI.00413-19 PMC6675881 31167921

[136] Tsai TT, Chen CL, Lin YS, Chang CP, Tsai CC, Cheng YL, Huang CC, Ho CJ, Lee YC, Lin LT, et al. Microglia retard dengue virus-induced acute viral encephalitis. Sci Rep. 2016;6:27670. 27279150 10.1038/srep27670 PMC4899773

[137] Camenga DL, Walker DH, Murphy FA. Anticonvulsant prolongation of survival in adult murine lymphocytic choriomeningitis. I. Drug treatment and virologic studies. J Neuropathol Exp Neurol. 1977;36:9–20. 833620 10.1097/00005072-197701000-00003

[138] Marker O, Nielsen MH, Diemer NH. The permeability of the blood-brain barrier in mice suffering from fatal lymphocytic choriomeningitis virus infection. Acta Neuropathol. 1984;63:229–39. 6464679 10.1007/BF00685249

[139] Kim JV, Kang SS, Dustin ML, McGavern DB. Myelomonocytic cell recruitment causes fatal CNS vascular injury during acute viral meningitis. Nature. 2009;457:191–5. 19011611 10.1038/nature07591 PMC2702264

[140] Guo Y, Chen J, Ji W, Xu L, Xie Y, He S, Lai C, Hou K, Li Z, Chen G, Wu Z. High-titer AAV disrupts cerebrovascular integrity and induces lymphocyte infiltration in adult mouse brain. Mol Ther Methods Clin Dev. 2023;31:101102. 37753218 10.1016/j.omtm.2023.08.021 PMC10518493

[141] Stewart-Hutchinson PJ. The pathogenic role of CD8 + T cells in experimental cerebral malaria. New York University. 2014.

[142] Nair AL, Groenendijk L, Overdevest R, Fowke TM, Annida R, Mocellin O, de Vries HE, Wevers NR. Human BBB-on-a-chip reveals barrier disruption, endothelial inflammation, and T cell migration under neuroinflammatory conditions. Front Mol Neurosci. 2023;16:1250123. 37818458 10.3389/fnmol.2023.1250123 PMC10561300

[143] Berndt P, Winkler L, Cording J, Breitkreuz-Korff O, Rex A, Dithmer S, Rausch V, Blasig R, Richter M, Sporbert A, et al. Tight junction proteins at the blood-brain barrier: Far more than claudin-5. Cell Mol Life Sci. 2019;76:1987–2002. 30734065 10.1007/s00018-019-03030-7 PMC11105330

[144] Cox B, Nicolai J, Williamson B. The role of the efflux transporter, P-glycoprotein, at the blood-brain barrier in drug discovery. Biopharm Drug Dispos. 2023;44:113–26. 36198662 10.1002/bdd.2331

[145] Sellers RS, Clifford CB, Treuting PM, Brayton C. Immunological variation between inbred laboratory mouse strains: points to consider in phenotyping genetically Immunomodified mice. Vet Pathol. 2012;49:32–43. 22135019 10.1177/0300985811429314

[146] Guglielmetti C, Levi J, Huynh TL, Tiret B, Blecha J, Tang R, VanBrocklin H, Chaumeil MM. Longitudinal imaging of T cells and inflammatory demyelination in a preclinical model of multiple sclerosis using (18)F-FAraG PET and MRI. J Nucl Med. 2022;63:140–6. 33837066 10.2967/jnumed.120.259325 PMC8717198

[147] Coolen T, Lolli V, Sadeghi N, Rovai A, Trotta N, Taccone FS, Creteur J, Henrard S, Goffard JC, Dewitte O, et al. Early postmortem brain MRI findings in COVID-19 non-survivors. Neurology. 2020;95:e2016–27. 32546654 10.1212/WNL.0000000000010116

[148] Fanizza F, Campanile M, Forloni G, Giordano C, Albani D. Induced pluripotent stem cell-based organ-on-a-chip as personalized drug screening tools: A focus on neurodegenerative disorders. J Tissue Eng. 2022;13:20417314221095339. 35570845 10.1177/20417314221095339 PMC9092580

[149] Inoue H, Park JH, Kiyotani K, Zewde M, Miyashita A, Jinnin M, Kiniwa Y, Okuyama R, Tanaka R, Fujisawa Y, et al. Intratumoral expression levels of PD-L1, GZMA, and HLA-A along with oligoclonal T cell expansion associate with response to nivolumab in metastatic melanoma. Oncoimmunology. 2016;5:e1204507. 27757299 10.1080/2162402X.2016.1204507 PMC5048759

[150] Skadborg SK, Maarup S, Draghi A, Borch A, Hendriksen S, Mundt F, Pedersen V, Mann M, Christensen IJ, Skjoth-Ramussen J, et al. Nivolumab reaches brain lesions in patients with recurrent glioblastoma and induces T-cell activity and upregulation of checkpoint pathways. Cancer Immunol Res. 2024;12:1202–20. 38885356 10.1158/2326-6066.CIR-23-0959 PMC11369628

[151] Liu L, Huang D, Matsui M, He TT, Hu T, Demartino J, Lu B, Gerard C, Ransohoff RM. Severe disease, unaltered leukocyte migration, and reduced IFN-gamma production in CXCR3-/- mice with experimental autoimmune encephalomyelitis. J Immunol. 2006;176:4399–409. 16547278 10.4049/jimmunol.176.7.4399

[152] Kohler RE, Comerford I, Townley S, Haylock-Jacobs S, Clark-Lewis I, McColl SR. Antagonism of the chemokine receptors CXCR3 and CXCR4 reduces the pathology of experimental autoimmune encephalomyelitis. Brain Pathol. 2008;18:504–16. 18422759 10.1111/j.1750-3639.2008.00154.x PMC8095644

[153] Lin J, Xu Y, Guo P, Chen YJ, Zhou J, Xia M, Tan B, Liu X, Feng H, Chen Y. CCL5/CCR5-mediated peripheral inflammation exacerbates blood–brain barrier disruption after intracerebral hemorrhage in mice. J Transl Med. 2023;21:196. 36918921 10.1186/s12967-023-04044-3 PMC10015963

[154] Wu Q, Xia Y, Xiong X, Duan X, Pang X, Zhang F, Tang S, Su J, Wen S, Mei L, et al. Focused ultrasound-mediated small-molecule delivery to potentiate immune checkpoint Blockade in solid tumors. Front Pharmacol. 2023;14:1169608. 37180717 10.3389/fphar.2023.1169608 PMC10173311

[155] Shan H, Zheng G, Bao S, Yang H, Shrestha UD, Li G, Duan X, Du X, Ke T, Liao C. Tumor perfusion enhancement by focus ultrasound-induced blood-brain barrier opening to potentiate anti-PD-1 immunotherapy of glioma. Transl Oncol. 2024;49:102115. 39217852 10.1016/j.tranon.2024.102115 PMC11402623

[156] Habib S, Singh M. Angiopep-2-Modified nanoparticles for Brain-Directed delivery of therapeutics: A review. Polym (Basel). 2022;14. 10.3390/polym14040712 PMC8878382 35215625

[157] Prabowo AS, Iyer AM, Anink JJ, Spliet WG, van Rijen PC, Aronica E. Differential expression of major histocompatibility complex class I in developmental glioneuronal lesions. J Neuroinflammation. 2013;10:12. 23347564 10.1186/1742-2094-10-12 PMC3565983

[158] Gupta A, Comfort B, Young K, Montgomery R. A pilot study to assess blood-brain barrier permeability in long COVID. Brain Imaging Behav. 2024;18:830–4. 38520594 10.1007/s11682-024-00877-8 PMC12205970

[159] Fujimoto T, Erickson MA, Banks WA. Neurotropism and blood-brain barrier involvement in COVID-19. Front. Drug Deliv. 2022;2:1073815.

[160] Trevino TN, Fogel AB, Otkiran G, Niladhuri SB, Sanborn MA, Class J, Almousawi AA, Vanhollebeke B, Tai LM, Rehman J, et al. Engineered Wnt7a ligands rescue blood-brain barrier and cognitive deficits in a COVID-19 mouse model. Brain. 2024;147:1636–43. 38306655 10.1093/brain/awae031 PMC11068107

[161] Greene C, Connolly R, Brennan D, Laffan A, O’Keeffe E, Zaporojan L, O’Callaghan J, Thomson B, Connolly E, Argue R, et al. Blood-brain barrier disruption and sustained systemic inflammation in individuals with long COVID-associated cognitive impairment. Nat Neurosci. 2024;27:421–32. 38388736 10.1038/s41593-024-01576-9 PMC10917679

