# Supplementary material for: Cytotoxic T lymphocytes and their dual role in modulating blood-brain barrier integrity in immune-mediated neurological pathologies
Source: J Transl Med. 2025 Dec 23;24:216. doi: 10.1186/s12967-025-07288-3 (PMC12903341; doi:10.1186/s12967-025-07288-3)
Supplement: Supplementary file 1 — Supplementary Material 1 [file 12967_2025_7288_MOESM1_ESM.docx]

**Supplementary Table 1 The discovery process of the BBB**

| **Name** | **Year** | **Contribution** | **Reference** |
| --- | --- | --- | --- |
| Ridley Humphrey | 1695 | The low permeability of small cerebral vessels | [1] |
| Ehrlich Paul | 1885 | The isolating between brain and bloodstream | [2] |
| Lewandowsky Max | 1909 | Terming this new concept as a German name bluthirnschranke | [3] |
| Goldmann Edwin Ellen | 1909 | Only the brain and the spinal cord can be stained by Evans blue injected in ventricles | [4] |
| Stern Lina & Raymond Gautier. | 1921 | Naming it as “barrière hémato-encéphalique” in French, and then translated into BBB | [5] |
| Stern Lina | 1929 | the BBB was not mature during embryogenesis | [6] |

**Supplementary Table 2 The main functions of components of BBB**

| **Components** | **Functions** | **Reference** |
| --- | --- | --- |
| Endothelial cells | Endothelial cells are tightly interconnected, forming distinct lumenal and abluminal membrane compartments | [7] |
| Pericytes | Pericytes are embedded in the basement membrane and lie abluminal to the endothelial cells, and in close communicate with endothelial cells | [8, 9] |
| Astrocytes | Astrocytes surround blood vessels in the brain, serving as the interface between neurons and endothelial cells | [10] |
| Tight junctions | Tight junctions reside between endothelial cells, serving as the main functional components in sustaining the permeability barrier and controlling tissue homeostasis | [11] |
| Adherent junctions | Adherent junctions are fundamental for the integrity of BBB, any change of adherens junctions may disrupt inter-endothelial cell connections | [12] |

**Supplementary Table 3 The main functions of immune checkpoints**

| **Checkpoints** | **Functions** | **Reference** |
| --- | --- | --- |
| PD-1 | Binding with its ligand PD-L1/PD-L2 of target cells, counteracting CD80–CD28 signaling transduction of CTLs. | [13] |
| CTLA-4 | Interferes with CD8 T-cell movements and the ability to form stable conjugates with APCs, thus reducing the contact time between cells | [14] |
| LAG-3 | Binding with CD3 in the TCR complex and inhibiting its signal transduction, leading to reduced T cell proliferation and cytokine production | [15] |
| TIM-3 | The switching of the binding TIM-3 and Bat3 or Fyn, further inhibiting upstream TCR signaling | [16] |
| TIGIT | Inhibiting TCR signaling by binding with CD155 of APCs | [17] |
| ICOS | Weaking the function of CD28 signaling by binding with CD275 of APCs | [18] |

**Reference**

1. Ridley H: *The Anatomy of the Brain. Printers to the Royal Society.* 1695.

2. Ehrlich P: *Das Sauerstoff-Bedürfniss des Organismus: eine farbenanalytische Studie.* A. Hirschwald; 1885.

3. Lewandowsky MJZkM: **Zur lehre der cerebrospinalflussigkeit.** 1909, **40:**480-494.

4. Goldmann EE: *Die äussere und innere skeretion des Gesunden organismus im lichte der "Vitalen Färbung".* H. Laupp'schen Buchhandlung; 1909.

5. Stern L, Gautier R: **Recherches sur Le liquide céphalo-rachidien: I.–Les rapports entre Le liquide céphalo-rachidien et la circulation sanguine.** *Archives Internationales de Physiologie* 1921, **17:**138-192.

6. Stern L, Rapoport J, Lokschina EJCSB: **Le fonctionnement de la barrière hémato-encéphalique chez les nouveau nés.** 1929, **100:**231-223.

7. Betz AL, Firth JA, Goldstein GW: **Polarity of the blood-brain barrier: distribution of enzymes between the luminal and antiluminal membranes of brain capillary endothelial cells.** *Brain Res* 1980, **192:**17-28.

8. Frank RN, Turczyn TJ, Das A: **Pericyte coverage of retinal and cerebral capillaries.** *Invest Ophthalmol Vis Sci* 1990, **31:**999-1007.

9. Armulik A, Genove G, Mae M, Nisancioglu MH, Wallgard E, Niaudet C, He L, Norlin J, Lindblom P, Strittmatter K, et al: **Pericytes regulate the blood-brain barrier.** *Nature* 2010, **468:**557-561.

10. Zhao Z, Nelson AR, Betsholtz C, Zlokovic BV: **Establishment and Dysfunction of the Blood-Brain Barrier.** *Cell* 2015, **163:**1064-1078.

11. Tsukita S, Furuse M, Itoh M: **Multifunctional strands in tight junctions.** *Nat Rev Mol Cell Biol* 2001, **2:**285-293.

12. Turowski P, Kenny BA: **The blood-brain barrier and methamphetamine: open sesame?** *Front Neurosci* 2015, **9:**156.

13. Arasanz H, Gato-Canas M, Zuazo M, Ibanez-Vea M, Breckpot K, Kochan G, Escors D: **PD1 signal transduction pathways in T cells.** *Oncotarget* 2017, **8:**51936-51945.

14. Schneider H, Downey J, Smith A, Zinselmeyer BH, Rush C, Brewer JM, Wei B, Hogg N, Garside P, Rudd CE: **Reversal of the TCR stop signal by CTLA-4.** *Science* 2006, **313:**1972-1975.

15. Hannier S, Tournier M, Bismuth G, Triebel F: **CD3/TCR complex-associated lymphocyte activation gene-3 molecules inhibit CD3/TCR signaling.** *J Immunol* 1998, **161:**4058-4065.

16. Rangachari M, Zhu C, Sakuishi K, Xiao S, Karman J, Chen A, Angin M, Wakeham A, Greenfield EA, Sobel RA, et al: **Bat3 promotes T cell responses and autoimmunity by repressing Tim-3-mediated cell death and exhaustion.** *Nat Med* 2012, **18:**1394-1400.

17. Inozume T, Yaguchi T, Furuta J, Harada K, Kawakami Y, Shimada S: **Melanoma Cells Control Antimelanoma CTL Responses via Interaction between TIGIT and CD155 in the Effector Phase.** *J Invest Dermatol* 2016, **136:**255-263.

18. Wikenheiser DJ, Stumhofer JS: **ICOS Co-Stimulation: Friend or Foe?** *Front Immunol* 2016, **7:**304.
